# Phylogenomic analysis of the cystatin superfamily in eukaryotes and prokaryotes

**DOI:** 10.1186/1471-2148-9-266

**Published:** 2009-11-18

**Authors:** Dušan Kordiš, Vito Turk

**Affiliations:** 1 Department of Molecular and Biomedical Sciences, J. Stefan Institute, Ljubljana, Slovenia; 2 Department of Biochemistry and Molecular and Structural Biology, J. Stefan Institute, Ljubljana, Slovenia

## Abstract

**Background:**

The cystatin superfamily comprises cysteine protease inhibitors that play key regulatory roles in protein degradation processes. Although they have been the subject of many studies, little is known about their genesis, evolution and functional diversification. Our aim has been to obtain a comprehensive insight into their origin, distribution, diversity, evolution and classification in Eukaryota, Bacteria and Archaea.

**Results:**

We have identified *in silico *the full complement of the cystatin superfamily in more than 2100 prokaryotic and eukaryotic genomes. The analysis of numerous eukaryotic genomes has provided strong evidence for the emergence of this superfamily in the ancestor of eukaryotes. The progenitor of this superfamily was most probably intracellular and lacked a signal peptide and disulfide bridges, much like the extant Giardia cystatin. A primordial gene duplication produced two ancestral eukaryotic lineages, cystatins and stefins. While stefins remain encoded by a single or a small number of genes throughout the eukaryotes, the cystatins have undergone a more complex and dynamic evolution through numerous gene and domain duplications. In the cystatin superfamily we discovered twenty vertebrate-specific and three angiosperm-specific orthologous families, indicating that functional diversification has occurred only in multicellular eukaryotes. In vertebrate orthologous families, the prevailing trends were loss of the ancestral inhibitory activity and acquisition of novel functions in innate immunity. Bacterial cystatins and stefins may be emergency inhibitors that enable survival of bacteria in the host, defending them from the host's proteolytic activity.

**Conclusion:**

This study challenges the current view on the classification, origin and evolution of the cystatin superfamily and provides valuable insights into their functional diversification. The findings of this comprehensive study provide guides for future structural and evolutionary studies of the cystatin superfamily as well as of other protease inhibitors and proteases.

## Background

The cystatin superfamily consists of a large group of cystatin domain-containing proteins, most of which are reversible and tight-binding inhibitors of the papain (C1) and legumain (C13) families of cysteine proteases [1-4]. On the basis of sequence similarity, the presence or lack of disulfide bonds, and physiological localization, this superfamily has been divided in both mammals and birds into family 1 or stefins, family 2 or cystatins and family 3 or kininogens [5]. Subsequently, cystatins were divided into types 1, 2 and 3, based mainly on the number of cystatin domains [6]. Recently, another system of classifying peptidase (protease) inhibitors was introduced, based on similarities in protein sequences and three-dimensional (3D) structures. In this system, the cystatins are placed in family I25 that contains three subfamilies: I25A, B and C [7].

Cysteine protease inhibitors are widely distributed in metazoans and angiosperms. They can function to protect cells from unwanted proteolysis and to control intra- and extracellular protein degradation [8-10]. The role of these inhibitors in maintaining controlled proteolysis in humans is critical. Any deviation from controlled protein degradation may result in pathological processes including cancer, neurodegeneration, bone resorption and cardiovascular diseases [reviewed in [10-13]].

Studies of the plant protease papain and human cysteine cathepsins were crucial for the discovery of the cystatins. The isolation and characterization of the chicken egg-white protein inhibitor of the plant cysteine proteases ficin and papain [14], of the intracellular protein inhibitor of papain, cathepsins B and H from pig leucocytes and spleen [15] and from human epidermis [16] stimulated further studies of cysteine protease inhibitors. Soon after the discovery of these proteins, the name cystatin was introduced for chicken cysteine protease inhibitor, indicating its function [17]. Determination of the amino acid sequences of chicken cystatin [18] and human stefin A [19], the discovery that »γ-trace« protein [20] is a human cystatin C [18,21,22], and the discovery that the kininogens are inhibitors of cysteine proteases [23,24] were all crucial to the mapping of the cystatin superfamily. The vertebrate cystatins and stefins have been the focus of extensive research as regulators of proteolysis. As a result, a large body of information has been accumulated on the cystatin superfamily over the last two decades [3,4,25].

Although stefins and cystatins differ considerably in their amino acid sequences, their tertiary structures are conserved and exhibit the cystatin fold that is formed by a five stranded anti-parallel β-sheet wrapped around a five-turn α-helix [26,27]. The structure of the plant inhibitor oryzacystatin, determined by NMR spectroscopy, shows the same cystatin fold as the animal cystatins [28]. Mutagenesis, X-ray crystallography and NMR spectroscopy studies have identified three conserved regions in the cystatins and stefins that are important for their inhibition of papain-like cysteine proteases. These three regions include the N-terminal segment, the highly conserved region (QXVXG) that folds into a β-hairpin loop, and a second hairpin loop containing a similarly conserved segment (PW), all of which participate in the formation of a »wedge« that is complementary to the active site cleft of papain [26,27,29].

The evolutionary analyses of the cystatin superfamily took place in the pre-genomic era, from the mid 80 s [1,6,30] to the late 90 s [31]. Thus they were based on a small sample of taxonomic diversity and diversity within the cystatin superfamily. Since then, the number of representatives has increased significantly, largely due to the accumulation of mammalian [3,32,33] and plant [34] genomic sequences. Numerous proteins containing cystatin domains have been discovered that cannot be easily incorporated into the existing classification scheme, resulting in a growing problem of classifying the cystatin superfamily [32,33]. A large amount of protein sequence data for this superfamily therefore awaits comprehensive evolutionary classification.

Here we aim to obtain a comprehensive insight into the origin, distribution, diversity, evolution and classification of the cystatin superfamily in Eukaryota, Bacteria and Archaea. Such an analysis could not have been performed previously due to the small number of available completed eukaryotic and prokaryotic genomes. We traced the genesis and expansion of the cystatin superfamily through comparative genomic and phylogenomic analyses, using publicly available whole-genome information from more than 2100 prokaryotic and eukaryotic genomes, as well as from the numerous transcriptomic and proteomic databases. The results of this work underpin targeted functional and structural studies of the members of the cystatin superfamily.

## Methods

### Data mining

All database searches were performed online and were completed in May 2009. The databases analysed were the nonredundant (NR), EST, GSS, HTGS, WGS, as well as the microbial and eukaryotic genome databases at the National Center for Biotechnology Information (NCBI) http://www.ncbi.nlm.nih.gov. In addition, we searched the Ensembl http://www.ensembl.org and the Joint Genome Institute (JGI) http://www.jgi.doe.gov databases. Taxon-specific genome databases were searched through the ENSEMBL and JGI websites, while diverse taxon-specific transcriptomic databases were searched for all eukaryotic lineages at NCBI. To detect all the available representatives of cystatin superfamily, database searches were performed iteratively. Comparisons were performed using the TBLASTN program [35] with the E-value cutoff set to 10^-5 ^and default settings for other parameters. Diverse stefins, cystatins and cystatin domains have been used as queries. The Translate program http://www.expasy.org/tools/dna.html was used to translate DNA sequences. Attempts were made to identify novel representatives of the cystatin superfamily in the genomic and transcriptomic databases, as well as in diverse proteomic databases such as the specialized Merops database of proteases and their protein inhibitors http://merops.sanger.ac.uk and the general proteomic databases, such as Superfamily http://supfam.org, SMART http://smart.embl-heidelberg.de, PFAM http://pfam.janelia.org, InterPro http://www.ebi.ac.uk/interpro/, TreeFam http://www.treefam.org and Phylofacts http://phylogenomics.berkeley.edu/phylofacts/. The accession numbers of diverse eukaryotic representatives of the cystatin superfamily are available in additional files 1 and 2.

### Phylogenetic analysis

All the nonredundant eukaryotic and bacterial representatives of the cystatin superfamily have been included in the analyses. The cystatin domain in the newly discovered representatives of the cystatin superfamily was identified using the SMART, InterPro and Pfam domain databases. The protein sequences were aligned using Clustal W2 [36]. All the available correction models were tested, but the complex models were outperformed by the simple correction models. We therefore used uncorrected *p *distances for deduced amino acid sequences to measure the extent of sequence divergence. When analysing many divergent sequences and when the number of positions used is relatively small, the uncorrected distances are more effective for obtaining reliable topology than more complicated correction models, due to their smaller variance [37,38]. Phylogenetic trees were reconstructed using the neighbor-joining (NJ) method [39] and the maximum likelihood (ML) method [40]. The reliability of the resulting topologies was evaluated by 1000 bootstrap replications. Diverse bacterial and eukaryotic representatives of the cystatin superfamily were used as outgroups. Phylogenetic analyses were performed with the programs Treecon [41], MEGA 4.0 [42] and RAxML [40].

## Results and Discussion

### A large number of new and diverse representatives of the cystatin superfamily have been discovered in genomic, transcriptomic and proteomic databases

Although the cystatin superfamily is relatively well-represented in the Merops database [43], the data are limited mainly to mammals and plants. A much greater number of members of the cystatin superfamily are present in the general proteomic databases, such as Superfamily, PFAM, SMART, InterPro and TreeFam. Even in these databases, the majority of annotated proteins are either restricted to vertebrates and invertebrates (*e.g*., in the TreeFam), or are highly biased to the Metazoa and plants, due to the limited taxon sampling of the completed genomes.

To overcome these problems, all the publicly available genomic, transcriptomic and proteomic databases have been searched for new members of the cystatin superfamily. In the numerous annotated genome databases, some proteins have not yet been identified, some are not correctly annotated, and some have been incorrectly assembled. To find all the sequenced members of the cystatin superfamily we therefore used a phylogenomic analysis of publicly available eukaryotic and prokaryotic genomes and transcriptomes. The major problem of the EST transcriptomic database is the overrepresentation of the mammalian, vertebrate, and angiosperm species. We therefore limited searching of the EST database (using TBLASTN) to the numerous specific taxonomic groups. This approach was especially important in finding stefins and cystatins in a large number of diverse unicellular eukaryotes. We also used a PSI-BLAST search to find some divergent representatives. However, the analysis of the transcriptomic database provided the most divergent novel eukaryotic representatives of stefins and cystatins. The members are more conserved within the ancestral stefin lineage than in the ancestral cystatin lineage. The analysis of numerous bacterial and archaeal genomes has shown a surprisingly limited distribution of stefins and cystatins in only a few bacterial genomes.

### Two ancestral lineages of the cystatin superfamily, stefins and cystatins, exist in Eukaryota and Bacteria

An analysis of the phyletic distribution of the cystatin superfamily has shown the presence of only two ancestral lineages, stefins and cystatins, in eukaryotes and prokaryotes (Table 1). Because both lineages most probably duplicated prior to the divergence of the principal lineages of eukaryotes, they are referred to as ancestral or ancient eukaryotic paralogs.

**Table 1:** Phyletic distribution of the cystatin superfamily in the three domains of life

Taxonomic group	stefins	cystatins	multicystatins	bifunctional cystatins
**EUKARYOTA**	+	+	+	+
**EXCAVATA**	+	+	+	-
diplomonads	-	+	-	-
parabasalians	-	+	+	-
oxymonads	-	+	+	-
Euglenozoa	+	-	-	-
Heterolobosea	+	+	+	-
jakobids	+	+	-	-
Malawimonas	+	+	-	-
**SAR SUPERGROUP**	+	+	+	+
ciliates	+	+	+	-
Dinophyta	+	-	-	-
Perkinsus	+	-	-	-
Apicomplexa	-	-	-	-
diatoms	-	+	-	-
Blastocystis	+	-	-	-
Oomycetes	-	+	-	+
Eustigmatophyceae	+	-	-	-
Rhizaria	+	-	-	-
Haptophyta	+	+	-	-
Cryptophyta	+	-	-	-
**ARCHAEPLASTIDA**	-	+	+	+
Glaucophyta	-	+	-	-
red algae	-	-	-	-
green algae	-	+	-	+
land plants	-	+	+	+
**UNIKONTA**	+	+	+	+
Amoebozoa	+	-	-	-
Fungi	-	-	-	-
Choanozoa	+	+	-	+
Metazoa	+	+	+	+
**BACTERIA**	+	+	+	-
**ARCHAEA**	-	-	-	-

We found that stefins remained as a single gene or as small multigene families throughout the eukaryotes. In contrast to stefins, cystatins underwent more complex and dynamic evolution by numerous gene and domain duplications. Despite their similar gene and protein structures [3,4], stefins remained evolutionarily stable. Stable genes persist as a single copy over a wide range of distantly related species, whereas unstable genes undergo frequent duplication and loss in a process called birth-death evolution. In the cystatin superfamily stefins are present as a single copy genes in diverse eukaryotic species, while cystatin genes undergo active birth-death evolution across the same species. A significant unique feature of cystatins is their signal peptide, which is responsible for their extracellular targeting. The stefins lack the signal peptide and are intracellular inhibitors [3]. The name stefin, which was coined for the first cytoplasmic cysteine protease inhibitor of cysteine proteases [44], is used to clearly distinguish the cytoplasmic cysteine protease inhibitors from their secreted homologs with a single cystatin domain. Here, we provide an additional rationale for separate names by showing an early evolutionary divergence between these two lineages. In the Merops database, the stefins are limited mostly to mammals, a few vertebrates and very few invertebrates. Until now, the oldest known stefin was from the slime mold Dictyostelium [45]. In this study we found numerous new stefins in diverse eukaryotic lineages that provided new insights into their origin, distribution, diversity and evolution (Table 1).

In the early evolutionary studies of the cystatin superfamily there was much speculation concerning the nature of their ancestor [6]. It was proposed that the archetypal cystatin had no disulfide bridges and that, approximately 1 billion years ago (Bya), a precursor containing disulfide bridges appeared from which all subsequent disulfide bridge-containing cystatins evolved [6]. However, because cystatins and stefins are present in the majority of the eukaryotic supergroups and in some prokaryotes (Table 1), this widely accepted scheme of the evolution of the cystatin superfamily [6] needs to be revised.

The classification and evolution of the cystatin superfamily was inferred [6,30,31,46] in the pre-genomic era, based on comparison of a small sample of mostly mammalian sequences. Moreover, sequence divergence alone is often inadequate for inferring the origin and diversification of a protein superfamily. Even in the case of vertebrate and mammalian members of the cystatin superfamily, the level of sequence divergence between diverse families (*e.g*., cystatin *vs *stefin, CRES *vs *cystatin, cystatin *vs *kininogen and cystatin *vs *fetuin) is high. Therefore, by using only mammalian members, we can neither adequately classify the cystatin superfamily nor map the origin of its numerous orthologous families.

**Figure 1:**
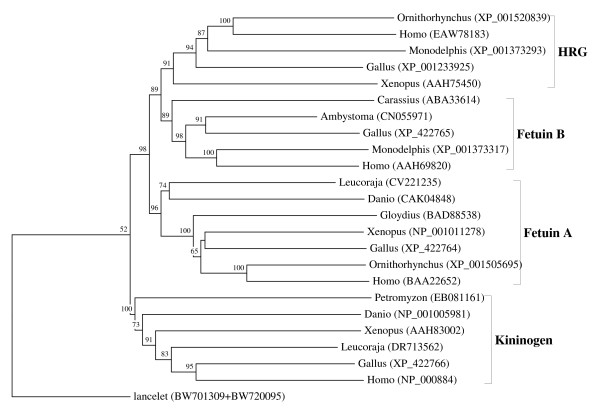
**Vertebrate multidomain representatives of the cystatin superfamily: kininogens, fetuins A and B and HRGs**. The rooted neighbor-joining tree shows the evolutionary relationships between the vertebrate-specific kininogen, fetuin and HRG orthologous families. NJ tree represents the bootstrap consensus following 1000 replicates, nodes with confidence values greater than 50% are indicated. Sequences were obtained from the GenBank, genus names and accession numbers are included.

The previously proposed evolutionary scheme assumed a near simultaneous origin and diversification of stefins, cystatins, kininogens and fetuins, occurring approximately 1 Bya [6]. However, only two ancestral lineages, stefins and cystatins, are present throughout the eukaryotes (Table 1), indicating that the above assumption is incorrect. The kininogens and fetuins are, in fact, much younger and restricted to the vertebrates (Figure 1; Table 2), and therefore originated, not 1 Bya, but at most 650 million years ago (Mya) (kininogens), 525 Mya (fetuin A) and 475 Mya (fetuin B) (divergence time estimates are from [47]). The early origin of multidomain cystatins, about 1 Bya, and their stepwise evolution were also proposed [6]. We demonstrated that the multidomain cystatins are not monophyletic, but originated independently by domain duplication, several times in diverse eukaryotic lineages (Table 1). The early models of the evolution of the multidomain cystatins [1,30] did not consider domain duplication as a major mechanism for their origin.

**Table 2:** Diversity of the cystatin superfamily in vertebrates

Orthologous family	Cyclostomata	Chondrichthyes	Actinopterygii	Amphibia	»Reptilia«	Aves	Prototheria	Metatheria	Eutheria
Stefin	+	+	+	+	+ (A + B)	+ (A + B)	+ (A + B)	+ (A + B)	+ (A + B)
Cystatin C	-	-	+	+	+	+	+	+	+
Cystatin E/M	-	+	-	-	+	-	+	+	+
Cystatin F	+	+	+	+	+	+	+	+	+
Fetuin A	-	+	+	+	+	+	+	+	+
Fetuin B	-	-	+	+	+	+	-	+	+
HRG	-	-	-	+	+	+	+	+	+
Kininogen	+	+	+	+	+	+	-	+	+
Cystatin 8	-	-	-	-	-	-	-	-	+
Cystatin 9	-	-	-	-	-	-	-	-	+
Cystatin 11	-	-	-	-	-	-	-	-	+
Cystatin 12	-	-	-	-	-	-	-	-	+
Cystatin 13	-	-	-	-	-	-	-	-	+
Cystatin 1L	-	-	-	-	-	-	-	-	+
Cathelicidin	+	+	+	+	+	+	+	+	+
Spp24	-	+	+	+	-	+	+	+	+
CRP1	-	-	-	-	-	-	-	-	+
Latexin	-	+	+	+	+	+	+	+	+
TIG1	-	-	-	+	+	+	+	+	+

In the earlier pre-genomic studies, the position of the plant cystatins was assumed to be unique, and they were regarded as structural intermediates between stefins and cystatins [6]. However, as our analysis shows, this assumption can no longer be maintained, because they represent only the cystatins present in the plant kingdom (Table 1).

Another long-standing assumption has been that the disulfide bridges have been conserved in cystatins since their first appearance [6]. While this conservation is apparent in mammals and vertebrates, it is less certain in the case of basal metazoans and plant cystatins. In the unicellular eukaryotes, the situation is quite complicated and no pattern of conservation can be recognized (additional file 3).

In summary, our analysis has revealed evidence against some of the generalizations and assumptions made in the pre-genomic era: (i) on the nature of the ancestor of the cystatin superfamily, (ii) on the classification and evolution of the cystatin superfamily, (iii) on the origin of and diversification of stefins, cystatins, kininogens and fetuins, (iv) on the unique position of plant cystatins and (v) on the conservation of the disulfide bridges in the cystatins.

### Distribution of the cystatin superfamily in the three domains of life

The phyletic distribution patterns of cystatins and stefins have been analyzed in Archaea, Bacteria and Eukaryota. Members of the cystatin superfamily have been found only in Eukaryota and in Bacteria; no members being present in Archaea (Table 1). Phylogenomic analysis indicates a relatively widespread distribution of the cystatin superfamily in eukaryotes. However, the distributions of two ancestral eukaryotic paralogous lineages (classes) in eukaryotes differ, particularly in their phyletic distribution (Table 1). Even in Bacteria, the phyletic distributions of cystatins and stefins are completely different, because cystatins are limited mainly to the Vibrios, while stefins show a wider, although still patchy distribution.

No cystatins or stefins have previously been reported from the genomes of the unicellular eukaryotes. However, by analysis of the rich transcriptomic data and the more limited genomic data for numerous unicellular eukaryotic lineages, large numbers of highly divergent cystatins and stefins were revealed (Table 1). These particular findings are crucial, since they provide a major insight into the origin and evolution of the cystatin superfamily, resulting in a new classification of the cystatin superfamily members.

Stefins are present in most major eukaryotic supergroups. In unikonts, they are present in Holozoa (Choanozoa and Metazoa) and Amoebozoa, but not in Fungi. They are absent from plant genomes but are present in diverse representatives of the SAR supergroup [48] - in alveolates, heterokonts, Rhizaria, Haptophyta and cryptomonads. Stefins are present in diverse representatives of Excavata, such as Euglenozoa, Heterolobosea, Jakobidae and Malawimonas, but not in the oxymonads, Trimastix, parabasalians or in diplomonads (Table 1).

Cystatins are more widely distributed in eukaryotic supergroups than stefins, being present in unikonts (in Holozoa only, but not in Fungi and Amoebozoa), in Plantae and in the SAR supergroup; however the phyletic distribution pattern is not the same as that for stefins. Cystatins are present in the diverse Excavata lineages, such as Heterolobosea, Jakobidae, Malawimonas, oxymonads, parabasalians and diplomonads (Table 1), where they are more abundant than stefins.

The phyletic distribution of the eukaryotic multidomain cystatins is limited. They are present in some of the Excavata lineages, such as parabasalians, oxymonads and heteroloboseans. In the SAR supergroup the multidomain cystatins are rare, being found in a few ciliates only. In the plant kingdom they are present in eudicots only. In unikonts, they were found in metazoans only, in both Protostomia (Ecdysozoa and Lophotrochozoa) and Deuterostomia (cephalochordates and vertebrates only) (Table 1). Phylogenomic analysis provides strong evidence that multidomain-cystatins are not monophyletic, but that they originated independently several times during the evolution of eukaryotes (Table 1).

Bifunctional cystatins simultaneously inhibit the C1 and C13 families of cysteine proteases [2]. Two separate lineages of bifunctional cystatins exist, one in metazoans, such as the vertebrate cystatins C, E/M and F [2] and a nematode cystatin [49], and the other in a few angiosperms [50]. Our analysis provides evidence that both lineages are more widespread in a number of older plant and metazoan lineages. Plant bifunctional cystatins occur in all land plant lineages and in a few green algae (additional file 4), while the metazoan bifunctional cystatins are widespread in eumetazoans (consisting of Cnidaria and Bilateria). Metazoan-type bifunctional cystatins were also found in a choanozoan *Monosiga ovata*, indicating their presence at least in Holozoa. Surprisingly, metazoan-type bifunctional cystatins are present in the oomycete *Phytophthora infestans*, where they are responsible for the inhibition of plant cysteine proteases [51].

We have analyzed in detail the distribution of cystatins in the plant kingdom (Archaeplastida) (additional file 4). They have previously been found only in a few angiosperm genomes [34]. We provide evidence that cystatins are present in the oldest lineage of the plant kingdom, in glaucophytes (additional file 4). In the genomes and transcriptomes of diverse green algae (Chlamydomonas, Volvox, Scenedesmus, Helicosporidium and Prototheca), we found highly divergent cystatins. Analysis of the rich collection of genome and EST data for diverse lineages of land plants demonstrated that cystatins are widespread in all major lineages, such as liverworts, mosses, lycophytes, ferns and all gymnosperm lineages, as well as in all angiosperm lineages (additional file 4). No cystatins can be found in the currently available genome and EST data from red algae.

The greatest diversity of stefins and cystatins is present in Metazoa. We found them in all major metazoan taxonomic groups, such as Porifera, Cnidaria, Ctenophora and in Bilateria (both in protostomes and deuterostomes) (additional file 5). No representatives of the cystatin superfamily can be found in the most compact metazoan genome, the *Trichoplax adherens *(Placozoa). Genomic analysis shows that sponges, cnidarians, protostomes and basal deuterostome lineages (xenoturbellids, echinoderms and urochordates) possess only cystatins and stefins (additional file 5). Multidomain cystatins are present in diverse protostome lineages such as ecdysozoans (in some arthropods) and lophotrochozoans, but in basal deuterostomes they are present only in cephalochordates (additional file 5). Phylogenomic analysis of the metazoan genomic and transcriptomic databases provides direct evidence that the major diversification inside the cystatin lineage has occurred several times during the evolution of the vertebrates and in the ancestor of placental mammals (Table 2).

### Evolutionary classification of the cystatin superfamily

The sequence data available in genomic, proteomic and transcriptomic databases can resolve the long-standing question of the classification and evolution of the cystatin superfamily. In the last decade, a number of novel mammalian cystatin representatives have been found and characterized, both biochemically and structurally [reviewed in [3,4,32] and [33]].

**Figure 2:**
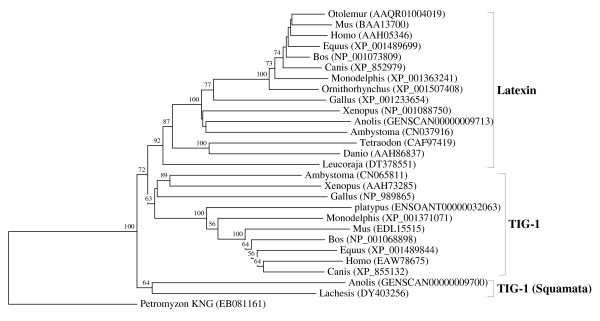
**Vertebrate-specific latexin and TIG1 orthologous families**. The rooted neighbor-joining tree shows the evolutionary relationships between the vertebrate-specific latexin and TIG1 orthologous families. NJ tree represents the bootstrap consensus following 1000 replicates, nodes with confidence values greater than 50% are indicated. Sequences were obtained from the GenBank and ENSEMBL, genus names and accession numbers are included.

**Figure 3:**
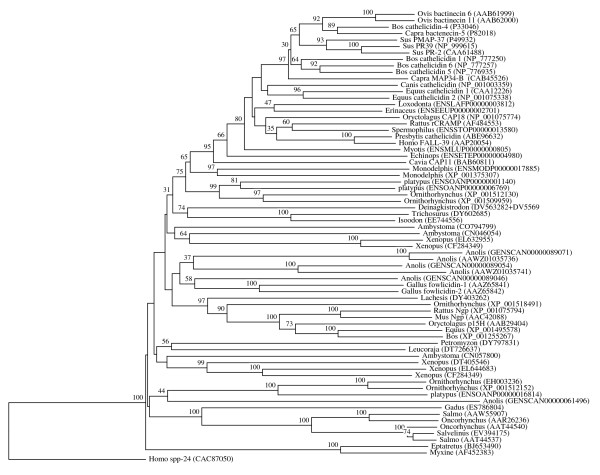
**Vertebrate-specific cathelicidin orthologous family**. The rooted neighbor-joining tree shows the evolutionary relationships inside the vertebrate-specific cathelicidin orthologous family. NJ tree represents the bootstrap consensus following 1000 replicates, nodes with confidence values greater than 30% are indicated. Sequences were obtained from the GenBank and ENSEMBL, genus names and accession numbers are included.

**Figure 4:**
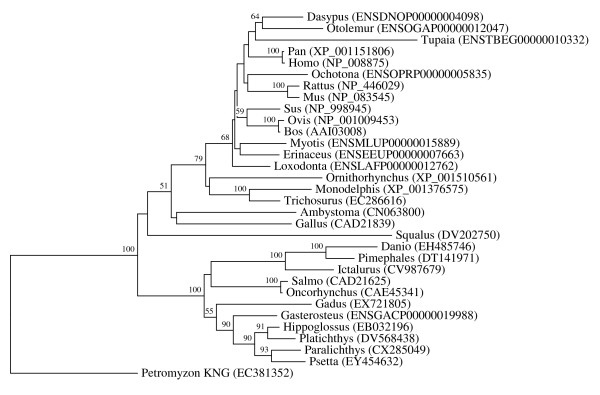
**Vertebrate-specific Spp24 orthologous family**. The rooted neighbor-joining tree shows the evolutionary relationships inside the vertebrate-specific Spp24 orthologous family. NJ tree represents the bootstrap consensus following 1000 replicates, nodes with confidence values greater than 50% are indicated. Sequences were obtained from the GenBank and ENSEMBL, genus names and accession numbers are included.

**Figure 5:**
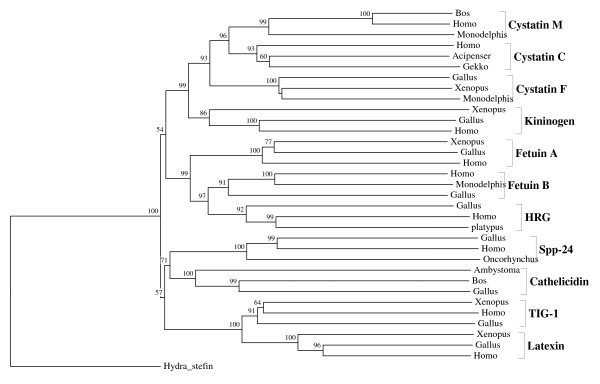
**Evolutionary relationships between the major vertebrate-specific orthologous families of the cystatin superfamily**. The rooted neighbor-joining tree shows the evolutionary relationships between the major vertebrate-specific orthologous families cystatins C, M and F, kininogens, fetuins A and B, HRGs, spp24s, cathelicidins, latexins and TIG1s. NJ tree represents the bootstrap consensus following 1000 replicates, nodes with confidence values greater than 50% are indicated. The following sequences were used in the reconstruction of the evolutionary relationships among diverse vertebrate orthologous families: **cystatins E/M: ***Bos taurus *(AAT46121), *Homo sapiens *(NP_001314) and *Monodelphis domestica *(XP_001379474); **cystatins C: ***Homo sapiens *(CAA36497), *Acipenser transmontanus *(DR975381) and *Gekko japonicus *(EB169380); **cystatins F: ***Gallus gallus *(XP_415013), *Xenopus tropicalis *(AAH88052) and *Monodelphis domestica *(XP_001382090); **kininogens: ***Xenopus laevis *(AAH83002), *Gallus gallus *(XP_422766) and *Homo sapiens *(NP_000884); **fetuins A:***Xenopus tropicalis *(NP_001011278), *Gallus gallus *(XP_422764) and *Homo sapiens *(BAA22652); **fetuins B: ***Homo sapiens *(AAH69820), *Monodelphis domestica *(XP_001373317) and *Gallus gallus *(XP_422765); **HRGs: ***Gallus gallus *(XP_001233925), *Homo sapiens *(EAW78183) and platypus (ENSOANP00000001023); **spp24s: ***Gallus gallus *(CAD21839), *Homo sapiens *(NP_008875) and *Oncorhynchus mykiss *(CAE45341); **cathelicidins: ***Ambystoma tigrinum *(CN057800), *Bos taurus *(NP_777250) and *Gallus gallus *(AAZ65842); **TIG1s: ***Xenopus laevis *(AAH73285), *Homo sapiens *(EAW78675) and *Gallus gallus *(NP_989865) and **latexins: ***Xenopus laevis *(NP_001088750), *Gallus gallus *(XP_001233654) and *Homo sapiens *(AAH05346). *Hydra magnipapillata *stefin (CV151842) has been used as an outgroup. Six Eutheria-specific CRES orthologous families and rodent-specific CRP1 family have not been included into the analysis, since they originated from the cystatin C.

**Figure 6:**
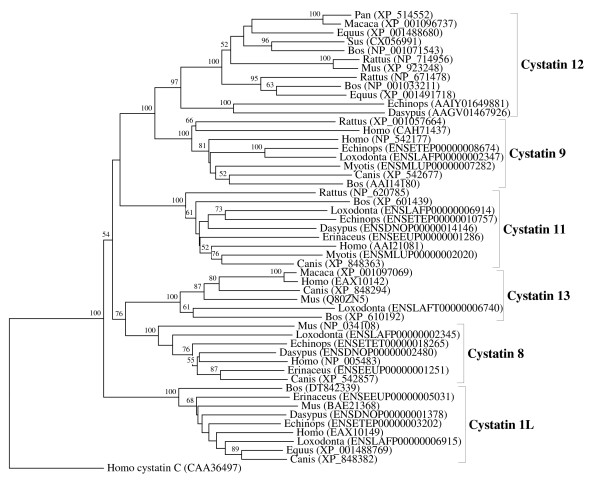
**Evolutionary relationships between the six Eutheria-specific orthologous families of the CRES subgroup of cystatins**. The rooted neighbor-joining tree shows the evolutionary relationships between the Eutheria-specific cystatin 12, cystatin 9, cystatin 11, cystatin 13, cystatin 8 and cystatin 1L orthologous families. NJ tree represents the bootstrap consensus following 1000 replicates, nodes with confidence values greater than 50% are indicated. Sequences were obtained from the GenBank and ENSEMBL, genus names and accession numbers are included.

**Figure 7:**
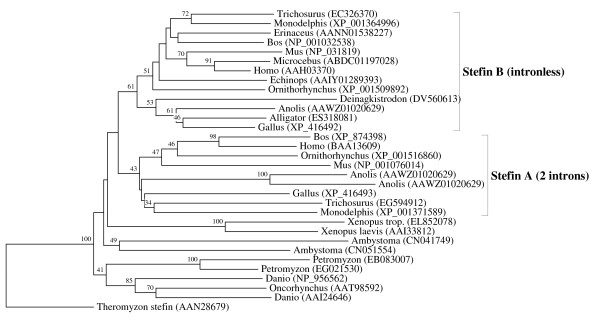
**Evolutionary relationships between the Amniota-specific stefin A and B orthologous families**. The rooted neighbor-joining tree shows the evolutionary relationships between the vertebrate-specific stefin A and B orthologous families. NJ tree represents the bootstrap consensus following 1000 replicates, nodes with confidence values greater than 30% are indicated. Sequences were obtained from the GenBank, genus names and accession numbers are included.

We performed numerous phylogenetic analyses, based on our large collection of cystatin superfamily representatives. They were analysed at the global level and at the level of particular taxonomic groups where great diversification has occurred (in angiosperms, vertebrates and mammals only), as well as at the level of particular orthologous families (mostly vertebrate-specific orthologous families: kininogens, fetuins A and B, HRG (Figure 1), latexins and TIG1 (Figure 2), cathelicidins (Figure 3), Spp24 (Figure 4), cystatins C, E/M and F (Figure 5), CRES subgroup (6 orthologous families) (Figure 6) and stefins A and B (Figure 7)). The NJ method with uncorrected distances was found to produce better resolution of evolutionary relationships in the cystatin superfamily than the more complex ML method. The reconstruction of evolutionary relationships in the cystatin superfamily from hundreds of protein sequences is difficult, because only a few sequence motifs are conserved. In the majority of previous studies of the cystatin superfamily, the reconstruction of evolutionary relationships was poor and attempts to classify them into monophyletic groups failed.

The protein sequences of cystatins and stefins are problematic for phylogenetic analysis, because they are quite short, highly divergent and produce low bootstrap values in the phylogenetic trees. We found that the reconstruction of evolutionary relationships in the cystatin superfamily is simple on the short evolutionary timescale (*e.g*., in mammals or in land plants), but it becomes difficult on the large evolutionary timescale. In the reconstruction of the evolutionary history and in classification in the cystatin superfamily, it is helpful that unicellular eukaryotes contain only two ancestral lineages, the stefins and cystatins (Table 1). The evolutionary relationships in the cystatin superfamily can easily be reconstructed for any particular taxonomic group (*e.g*., for angiosperms, mammals, vertebrates or bacteria), but is difficult for the unicellular eukaryotes (data not shown).

**Figure 8:**
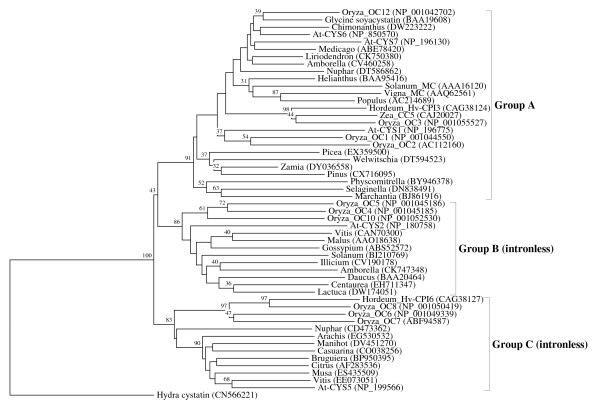
**Evolutionary relationships between the representatives of the cystatin superfamily in the land plants (Embryophyta)**. The rooted neighbor-joining tree shows the evolutionary relationships between the three orthologous families in the land plants. NJ tree represents the bootstrap consensus following 1000 replicates, nodes with confidence values greater than 30% are indicated. Sequences were obtained from the GenBank, genus names and accession numbers are included.

Phylogenomic analysis provided a rich collection of diverse representatives of the cystatin superfamily at different taxonomic levels (Tables 1 and 2; Figures 1, 2, 3, 4, 5, 6, 7 and 8; additional file 3). The analysis of numerous eukaryotic genomes has provided strong evidence for two ancient eukaryotic paralogous lineages, stefin and cystatin classes (Table 1). Inside the stefin lineage (class), there has been little functional diversification. A number of highly divergent orthologous families can be recognized only in the cystatin class, in a few multicellular eukaryotic lineages. Numerous orthologous families occur in vertebrates and mammals (20 families) and a few in angiosperms (3 families only) (Table 3; Figures 1, 2, 3, 4, 5, 6, 7 and 8; additional file 6). In all other taxonomic groups, only stefins and cystatins (including multidomain cystatins) can be found (Table 1). Due to the limited taxonomic sampling and their highly divergent sequences, the recognition of orthologous families in unicellular eukaryotes (if they exist) is currently not possible.

**Table 3:** Orthologous families in the cystatin superfamily

Orthologous family name	distribution	origin	lineage-specific expansion	number of cystatin domains
Stefin A	Amniota	LCA of Amniota	rodents	1
Stefin B	Amniota	LCA of Amniota	no	1
Cystatin C	Euteleostomi	LCA of Euteleostomi	primates	1
Cystatin F	Vertebrata	LCA of Vertebrata	no	1
Cystatin E/M	Gnathostomata	LCA of Gnathostomata	no	1
Spp24	Gnathostomata	LCA of Gnathostomata	no	1
Cathelicidin	Vertebrata	LCA of Vertebrata	Laurasiatheria	1
Cystatin 8	Eutheria	LCA of Eutheria	no	1
Cystatin 9	Eutheria	LCA of Eutheria	no	1
Cystatin 11	Eutheria	LCA of Eutheria	no	1
Cystatin 12	Eutheria	LCA of Eutheria	no	1
Cystatin 13	Eutheria	LCA of Eutheria	no	1
Cystatin 1L	Eutheria	LCA of Eutheria	no	1
CRP1	Muridae	LCA of Muridae	Muridae	1
Fetuin A	Gnathostomata	LCA of Gnathostomata	no	2
Fetuin B	Euteleostomi	LCA of Euteleostomi	no	2
HRG	Tetrapoda	LCA of Tetrapoda	no	2
Kininogen	Vertebrata	LCA of Vertebrata	no	3
Latexin	Gnathostomata	LCA of Gnathostomata	no	1 or 2
TIG1	Tetrapoda	LCA of Tetrapoda	no	1 or 2
Group A	Viridiplantae	LCA of Viridiplantae	Magnoliophyta	from 1 to multiple
Group B	Magnoliophyta	LCA of Magnoliophyta	no	1
Group C	Magnoliophyta	LCA of Magnoliophyta	no	1

We uncovered a problem in automated protein subfamily identification and classification, for example in PhyloFacts (phylogenomics.berkeley.edu/phylofacts/), by which no less than 42 subfamilies were documented inside the cystatin superfamily. The major problem of the automated protein subfamily identification and classification approach originates from limited taxon sampling (restricted to the metazoans and angiosperms only) and resulted in assigning the status of a subfamily to every single divergent sequence.

### Phylogenomic analysis of the cystatin superfamily

#### Origin of the cystatin superfamily

Phylogenomic analysis of the cystatin superfamily in all three domains of life has provided strong evidence for their origin prior to the divergence of the principal eukaryotic lineages. The ancestor of this superfamily was most probably intracellular, lacking a signal peptide and disulfide bridges, similar to the extant Giardia cystatin (additional file 7). The result of the primordial gene duplication in the ancestor of eukaryotes was the emergence of the two ancestral eukaryotic lineages, cystatins and stefins. The analysis of all eukaryotic orthologous families provides strong evidence about their origins (*i.e*., where and when they originated and from which progenitor). Only a few cases of bursts in functional diversification have occurred, in some of the multicellular eukaryotes, one in land plants (angiosperms), the second during evolution of the vertebrates, and a third in the ancestor of placental mammals.

In the case of land plants, the origin of the ancestral cystatin lineage A can be traced, at least in the ancestor of land plants (Figure 8). This orthologous family is widespread in land plants, present in liverworts, mosses, lycophytes, ferns, gymnosperms and in angiosperms. Green algal and glaucophyte representatives cannot provide phylogenetic evidence for their inclusion into the ancestral lineage A. The problem is that algal and glaucophyte representatives are highly divergent, therefore their position is at the base of the tree. Yet, in the most ancestral plant lineages (glaucophytes and green algae), only a single or a few cystatin genes can be found, indicating that the ancestral land plant specific phytocystatin lineage A is a direct descendant of these much older algal cystatins. The age of this phytocystatin lineage is therefore between 450 My and 1.5 By (if this lineage originated in the LCA of the plant kingdom). We found that most representatives in the ancestral cystatin lineage A, from land plants and a few green algae, possess the highly conserved legumain binding motif (SNSL) in the C-terminal part of the molecule [50] (additional file 8). This finding strongly connects the green algal and land plant cystatins, and provides direct evidence that the ancestral plant cystatin lineage A originated about 1-1.5 Bya, at least in the LCA of the green plants (Viridiplantae), but probably even earlier in the LCA of the Archaeplastida (consisting of the land plants, green and red algae and the glaucophytes). The two much younger phytocystatin lineages (B and C) emerged in the LCA of angiosperms about 170 - 200 Mya (Figure 8). These novel lineages B and C are highly divergent, therefore they most probably evolved new biological functions. In contrast to the younger phytocystatin lineages B and C, we observed a rich evolutionary record of functional diversification in the ancestral land plant specific cystatin lineage A. Functional diversification in lineage A originated recently (~100 Mya or younger) and is limited to angiosperms (to some monocots and eudicots): in the eudicot-specific multicystatin subfamily, in the monocot-specific subfamily (OC-3) and in the bifunctional cystatins (OC-12) (Figure 8).

In contrast to the relatively low level of functional diversification of cystatins in plants, diversification was much greater during the evolution of vertebrates and in the ancestor of placental mammals (Table 2). Analysis of the cystatin superfamily in metazoans (additional file 5) has demonstrated that the basal deuterostomes (origin about 900 Mya) possessed only single copies of the cystatin and stefin genes. The ancestor of chordates also possessed single stefin and cystatin genes, but in cephalochordates the multidomain cystatins originated independently (additional file 5). The first diversification of cystatins occurred in the ancestor of vertebrates when the first four orthologous families emerged: cystatin F, cathelicidin, the progenitor of kininogen, and the progenitors of cystatins C and E/M. Their age is about 650 My. These first vertebrate orthologous families originated from the ancestral chordate cystatin and have remained stable in all vertebrates (Table 2; Figures 1, 3 and 5). Only a few novel orthologous families emerged in the ancestor of jawed vertebrates (Gnathostomata): fetuin A, spp24, cystatin E/M and latexin (Table 2; Figures 1, 2, 4 and 5). In the ancestor of bony vertebrates (Euteleostomi), two novel orthologous families originated: fetuin B and cystatin C (Table 2; Figures 1 and 5). In the ancestor of land vertebrates (Tetrapoda), two novel orthologous families appeared: TIG1 and HRG (Table 2; Figures 1 and 2). Six orthologous families originated in the ancestor of placental mammals (Eutheria), (Table 2; Figure 6) and constitute the CRES subgroup of cystatins [32]. These orthologous families are cystatins 8, 9, 11, 12, 13 and 1L (Table 2; Figure 6). Duplication of the stefin gene in the ancestor of the Amniota produced two orthologous families, stefins A and B (Table 2; Figure 7). They differ in their expression profiles and tissue specificities [3]. A few smaller and evolutionarily younger orthologous families and subfamilies have emerged in some mammalian orders, such as the Muridae-specific CRP1 [52], and a primate-specific subfamily of salivary cystatins (cystatins S, SA, SN and D) [53].

The number of orthologous families inside the cystatin superfamily differs between the diverse vertebrate classes (Table 2). In cyclostomes, besides the stefin, only four cystatin-derived orthologous families are present, while the genomes of the jawed vertebrates possess from 7 to 17 (18 in rodents only) orthologous cystatin-derived families and stefin (Table 2). In cartilaginous fishes, there are seven orthologous cystatin-derived families and stefin and, in teleost fishes, eight (loss of cystatin E/M) orthologous cystatin-derived families and stefin (Table 2). In amphibians, we found ten cystatin-derived orthologous families (loss of cystatin E/M) and stefin (Table 2). It is interesting that all land vertebrates (Tetrapoda), from amphibians to the mammals, possess a similar number of orthologous families, which is approximately eleven (Table 2). Birds also lost the cystatin E/M gene, while the most ancestral extant mammals (Prototheria, represented here by platypus) have lost fetuin B and kininogen genes (Table 2). The greatest number of cystatin-derived orthologous families inside the cystatin superfamily is present in the genomes of placental mammals (Eutheria). They possess 17 orthologous cystatin-derived families, as well as stefins A and B. These orthologous families remained conserved from the oldest representatives of placental mammals (Atlantogenata, a mammal clade containing the superorders Afrotheria and Xenarthra) to the primates (Table 2). Within the mammals, a large difference between placental (Eutheria) and ancestral (Prototheria and Metatheria) mammals is clearly evident, because the latter possess only 11 orthologous cystatin-derived families in addition to stefins A and B (Table 2).

The emergence of the vertebrate cystatin-derived orthologous families (Table 2; Figures 1, 2, 3, 4, 5, 6, and 7) is most probably connected to the origin of the adaptive immune system, the bone skeleton and the expansion of the innate immune system. The origins of cystatin-derived orthologous families in placental mammals (Figure 6) and the evolutionarily younger mammalian cystatin lineages are connected to the expansion of the innate immune system and are involved in host defence in specialized tissues, such as the CRES subgroup of cystatins [32] in the male gonads of placental mammals and the salivary cystatins in the salivary glands of primates [53].

#### Functional diversification

Phylogenomic analysis of the cystatin superfamily (Tables 1, 2 and 3; Figures 1, 2, 3, 4, 5, 6, 7 and 8; additional files 3, 4, 5, 6, 7 and 8), together with previous studies [3,32-34], has demonstrated that functional diversification in the cystatin lineage occurred only in a few multicellular eukaryotic lineages (Table 3).

The neofunctionalization in the cystatin superfamily occurred very early, as indicated by differences in the gene structure and expression profiles of the two ancestral lineages. We found an important aspect of division of labour in the cystatin superfamily. While stefins remained intracellular proteins, responsible for the regulation of endogenous protein turnover, the cystatins gained a signal peptide and emerged as extracellular inhibitors, responsible for the regulation of the exogenous protein turnover. Therefore one of the ancestral function of stefins may have been the inhibition of endogenous, and of cystatins the inhibition of exogenous, cysteine proteases. Eukaryotic cystatins are indeed defence- or attack-related proteins involved in the host-pathogen/parasite/predator interactions. Since the vast majority of eukaryotes are unicellular organisms, the origin of two ancestral cysteine protease inhibitor lineages with specialized functions may be very important for their hosts. For some pathogenic unicellular eukaryotes, such as for oomycetes, the role of cystatins in pathogen attack of the plant host has been demonstrated [51]. It is also interesting that the intracellular stefins are more conserved than the very divergent extracellular cystatins, as reported for some other proteins [54].

In plants, most cystatins function as inhibitors of both endogenous and exogenous cysteine proteases. It appears that, in the case of several taxonomic groups, just as the plants lost the stefin lineage, the cystatin lineage has gained an additional function - the inhibition of endogenous (intracellular) cysteine proteases. We found that among the unicellular eukaryotic lineages (Tables 1, 2 and 3; Figures 1, 2, 3, 4, 5, 6, 7 and 8; additional files 3, 4, 5, 6, 7 and 8), there are numerous examples of loss of one or both ancestral lineages (additional file 6). We expect that, in these organisms or in the whole taxonomic groups, the remaining cystatin or stefin lineage has gained an additional function, the inhibition of endogenous or exogenous cysteine proteases. It is interesting that in the case of several unicellular eukaryotic stefins (e.g. in Hyperamoeba, Capsaspora and Karlodinium) the newly gained signal peptide can be found, which is absent from the vast majority of metazoan and eukaryotic stefins (additional file 9). By acquiring the signal peptide, as observed in some unicellular eukaryotic stefins, they could gain a novel host defence-related function. In the plants there are two well known examples of the loss of ancestral function and gain of a novel function. One is monellin, an intensely sweet protein [55], and the other SQAPI, a Cucurbitales-specific cystatin being recruited as a protease inhibitor of aspartic proteases [56].

In vertebrates, ancestral inhibitory function has been retained in cystatins C, E/M and F, in kininogens and in stefins. Some of them gained specialized tissue-specific activity, such as cystatins E/M and F which inhibit some cell type-, tissue- or pathogen-specific cysteine proteases [3,32,33,53]. In the majority of vertebrate orthologous families the prevalent trend was loss of the ancestral inhibitory activity and acquisition of a novel function. This has been well documented in the following vertebrate orthologous families: fetuins A and B [57], HRG [58], cathelicidins [59,60], latexins and TIG1 [61], and spp24 [62], as well as in the mammalian families - in CRP1 [52] and in the six orthologous families of the CRES subgroup [32]. While these orthologous families still possess the conserved cystatin domain, they have lost inhibitory activity due to mutations in structurally important regions. Most of the above mentioned orthologous families are still involved in innate immunity [32,57-59,61]. HRG, latexin and cathelicidin gained antimicrobial activity [58-61] and HRG became also the inhibitor of angiogenesis [58]. Fetuins A and B, spp24 and latexin are reported to be involved in bone regulation and calcification [57,61,62]. Change of the inhibitory class has also occurred several times - at least some of the CRES cystatins inhibit serine protease prohormone convertase 2 [63], latexin inhibits zinc-dependent metallocarboxypeptidases (carboxypeptidase A4) [61], HRG inhibits thrombospondin-1 [58] and fetuin A is a binding partner for calpain domain III [57]. The number of newly gained functions in the vertebrate orthologous families indicates that the cystatin domain is a very diverse protein-protein interaction module that can readily interact with novel targets [3,32,33,53].

#### Gene loss

We found that stefins and cystatins have been lost from many eukaryotic genomes or from whole taxonomic groups (additional file 6). Gene loss in the cystatin superfamily can be quite easily recognized, since we inferred a eukaryotic ancestral state for this superfamily that is the presence of cystatins and stefins in the same genome. We also inferred the ancestral and derived states for all eukaryotic supergroups (additional file 10). The demonstration of the ancestral state for the cystatin superfamily is important for the recognition of several independent cases of gene loss of stefins, cystatins or of both lineages in diverse eukaryotic taxonomic groups (additional file 6). The evidence for all these gene losses is based on the analysis of complete genomes and not on partial EST data. Some taxonomic groups with very large genome data coverage (such as fungi, where ~100 genomes are finished) have lost both stefins and cystatins. These large taxonomic groups are Fungi, Kinetoplastida and Apicomplexa (additional file 6). In a number of eukaryotic pathogens, we observed complete loss of cystatin superfamily representatives (additional file 6); some of them use horizontally acquired bacterial chagasins for self-defence, attack or the regulation of proteolysis [64]. This surprising loss of cystatins and stefins from some eukaryotic pathogens indicates that they are not essential for them.

#### Horizontal gene transfer (HGT)

During the analysis of the cystatin superfamily we found no evidence of HGT for cystatins and stefins among the eukaryotic hosts. However, a rare case of horizontal transfer of cystatin gene was found from an insect host (ichneumonid wasp) to the symbiotic virus (Bracovirus, Polydnaviruses) of hymenopteran insects [65]. Strong evidence for HGT of stefins and cystatins was found in bacteria, and is described below.

### The cystatin superfamily in prokaryotes

#### Extremely limited distribution of cystatin superfamily in prokaryotes

In this study we demonstrated, for the first time, the presence of cystatin superfamily representatives in bacterial genomes. Despite the availability of a very large number of finished and unfinished bacterial and archaeal genomes - at the NCBI microbial genome database their number is 1823 - stefins and cystatins were found in a surprisingly small number of genomes (Table 4). Further, in the 68 archaeal genomes analyzed, neither stefins nor cystatins were found. The observed distribution of stefins and cystatins in bacterial genomes is therefore very limited and patchy. Mapping the presence and absence of cystatins and stefins in the 25 bacterial phyla revealed that cystatins are present in only two, and stefins in eight phyla, in which they are not widespread (Table 4).

**Table 4:** Phyletic distribution of the cystatin superfamily in Archaea and Bacteria

Phylum/class	stefins	cystatins
**ARCHAEA**	-	-
Euryarchaeota	-	-
Crenarchaeota	-	-
Nanoarchaeota	-	-
**BACTERIA**	+	+
***Hydrobacteria***	+	+
Proteobacteria	+	+
class Alphaproteobacteria	-	-
class Betaproteobacteria	+	-
class Gammaproteobacteria	+	+
class Deltaproteobacteria	+	+
class Epsilonproteobacteria	-	-
Bacteroidetes	+	+
Chlorobi	+	-
Chlamydiae	-	-
Planctomycetes	-	-
Spirochaetes	+	-
Acidobacteria	-	-
***Terrabacteria***	+	-
Cyanobacteria	+	-
Chloroflexi	-	-
Firmicutes	+	-
class Clostridia	+	-
class Mollicutes	-	-
class Bacilli	-	-
Actinobacteria	+	-
Deinococcus/Thermus	-	-
***Unclassified bacterial phyla***	-	-
Thermodesulfobacteria	-	-
Chrysiogenetes	-	-
Thermomicrobia	-	-
Nitrospira	-	-
Deferribacteres	-	-
Verrucomicrobia	-	-
Fibrobacteres	-	-
Dictyoglomi	-	-
Gemmatomonadetes	-	-
Lentisphaerae	-	-
***Basal bacterial phyla***	+	-
Fusobacteria	+	-
Aquificae	-	-
Thermotogae	-	-

Bacterial phyla are listed in accord with the latest bacterial phylogeny [74].

**Figure 9:**
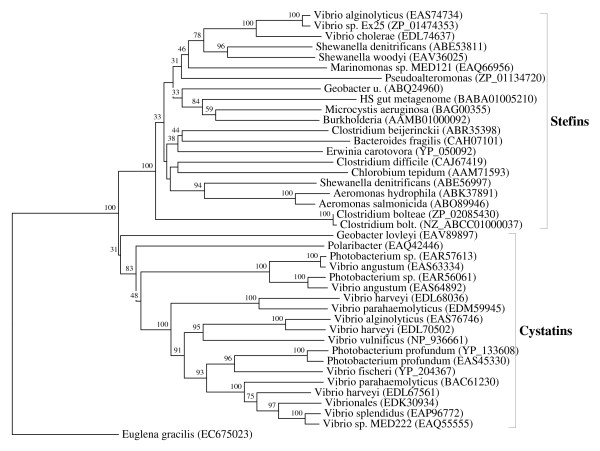
**Bacterial representatives of the cystatin superfamily**. The rooted neighbor-joining tree shows the evolutionary relationships between the bacterial cystatins and stefins. NJ tree represents the bootstrap consensus following 1000 replicates, nodes with confidence values greater than 30% are indicated. Sequences were obtained from the GenBank, species names and accession numbers are included.

Cystatins are present in the following bacterial phyla: in Bacteroidetes and in Proteobacteria (in the classes Gamma- and Delta-proteobacteria). They were found in four genera only: in Polaribacter, Vibrio, Photobacterium and Geobacter. These organisms inhabit quite diverse ecological habitats and niches. Some are pathogens of humans (*V. cholerae *and *V. vulnificus*), animal symbionts (*e.g. V. fischeri *in the squid) or deep sea bacteria (Photobacterium). Some are present in sediment microcosm (Geobacter) or are aerobic heterotrophic marine bacteria that adhere to the surfaces of other marine organisms (Polaribacter). In the case of the genus Geobacter seven genomes are available at the NCBI microbial genome database, but cystatin is present in only one (*G. lovleyi*). 44 genomes of the genus Vibrio are available at the NCBI microbial genome database and cystatins were found in 20, but not in the pathogen *V. cholerae *(Figure 9). Two genomes in the genus Polaribacter are available, and cystatin was found in one species, *P. dokdonensis*. Cystatins were found in all three available genomes in the genus Photobacterium. Phylogenetic analysis has shown that some Vibrio species possess small, diversified cystatin families that originated by gene duplication (Figure 9). The cystatin from Polaribacter (EAQ42446) is very interesting, since it shows the triplication of the cystatin domain and gene fusion with another gene.

Stefins are present in eight bacterial phyla: Bacteroidetes, Chlorobi, Cyanobacteria, Firmicutes (in the class Clostridia only), Proteobacteria (in the classes Beta-, Gamma- and Delta-proteobacteria), Actinobacteria, Spirochaetes and Fusobacteria. Their extremely limited and patchy distribution in bacterial genomes is striking, their being found in 17 bacterial genera only: Bacteroides, Chlorobium, Microcystis, Clostridium, Burkholderia, Aeromonas, Vibrio, Shewanella, Erwinia (Pectobacterium), Marinomonas, Pseudoalteromonas, Geobacter, Lutiella, Eggerthella, Oribacterium, Brachyspira and Fusobacterium. As in the case of bacterial cystatins, these organisms inhabit highly diverse habitats. Some are pathogens of animals (Clostridium, Aeromonas, *Vibrio cholerae *and Burkholderia, Brachyspira, Oribacterium, Eggerthella) and plants (Erwinia), some are marine bacteria (Marinomonas, Shewanella), while others are comensals in human gut microbiota (*Bacteroides fragilis*, *Fusobacterium varium*, *Clostridium asparagiforme*). Some are thermophilic bacteria living in hot springs (*Chlorobium tepidum*), while others are present in the sediments of uranium bioremediations (*Geobacter uraniumreducens *and Lutiella) or in soil (*Clostridium beijerinckii*); others are biofilm-forming marine bacteria on the surface of eukaryotic organisms (*Pseudoalteromonas tunicata*) or are widespread toxic bloom-forming cyanobacteria (*Microcystis aeruginosa*).

Not only is this list of habitats unusual and diverse, but the presence of stefins in the genomes of these genera is also highly restricted. The question is, how widespread is the distribution of stefins in these large congeneric collections of bacterial genomes. For several of the above mentioned genera a large number of genomes are available at the NCBI - up to 62 in the case of Burkholderia. However, their distribution in genomes of the congeneric species also proves to be extremely limited. In the genus Bacteroides 28 genomes are available at the NCBI, but stefins are present only in three, which in fact are strains of the same species. The stefin from *Bacteroides fragilis *(CAH07101) is very interesting, since it shows gene fusion with the chagasin gene. In the genus Chlorobium seven genomes are available and stefins are present in only one, *Chlorobium tepidum*. In the case of the genus Clostridium 60 genomes are available, but stefins are present in only 15. Even more striking is the sparse presence of stefins in the genus Burkholderia, for which no less than 62 genomes are available at the NCBI - stefins are present in only one. It is interesting that, in the genus Vibrio, stefins are present in 25 out of 44 available genomes. However, 23 of these genomes correspond to the 23 different strains of *Vibrio cholerae*. Shewanella genus is represented by 21 genomes at the NCBI, but stefins are present in only two. For the remaining bacterial species where stefins were found, from two to seven congeneric genomes are available and, overall, stefins were found in a very limited number only (Figure 9). This extensive and genome-wide analysis of the distribution of stefins and cystatins in bacterial genomes provides strong evidence for their very limited distribution (Figure 9; additional file 10).

Distribution of stefins and cystatins in particular phyla is also very interesting. Of 77 genomes available in the phylum Bacteroidetes, stefins were found in only three and cystatins in two distinct genomes. In the phylum Chlorobi (green sulphur bacteria) 12 genomes are available at the NCBI, but stefins were found in a single genome only. 54 genomes are available for the phylum Cyanobacteria at the NCBI, but stefins were found in only one. 160 genomes are available for the phylum Actinobacteria at the NCBI, but stefins were found in only one. Two other very large phyla, Firmicutes (458 genomes at the NCBI microbial genome database) and Proteobacteria (817 genomes at the NCBI microbial genome database), provide the vast majority of the currently available bacterial genomes, more than 1270 genomes, but despite such a large collection the distribution of stefins, (in 39 genomes out of 1275 available) and cystatins (in 19 genomes out of 1275 available), in these major bacterial phyla is extremely limited.

What is the reason for such a strange and patchy distribution of cystatin superfamily in bacterial genomes? A null hypothesis is that they were present in the LUCA, but have frequently been lost from their genomes. The major problem with this hypothesis is the patchy presence of cystatins and stefins in the densely sampled genomes of numerous congeneric species. In the case of very large bacterial phyla we have no distribution pattern that can provide evidence for the presence of cystatins and stefins in their ancestral lineages.

#### What are the functions of the newly acquired cystatins and stefins in bacterial genomes?

Cystatins and stefins are the natural inhibitors of the eukaryote-specific cysteine proteases, the cysteine cathepsins [3,4,9]. Since bacterial stefins and cystatins possess the same highly conserved structural motifs as those in eukaryotic organisms, they most probably inhibit the cysteine proteases of their eukaryotic hosts (additional file 11). On the basis of the conserved secondary and 3D structures (data not shown) we expect that the biochemistry of the bacterial stefins and cystatins will be similar to that of the eukaryotic ones, with possible new structural or functional roles regarding their activity, specificity and targeting. The vast majority of prokaryotic genomes contain very large numbers of C1 family of cysteine proteases and, since their natural inhibitors are chagasins [64], the newly gained cystatins and stefins are unlikely to have been acquired for the endogenous regulation of the bacterial cysteine proteases. Experimentally verified inhibition of the eukaryotic, but not bacterial, cysteine protease (cathepsin L) exists only for bacterial chagasin from *Pseudomonas aeruginosa *[66]. It was proposed that the bacterial chagasins may function as inhibitors of their own or of the host cysteine proteases (as a novel virulence factors) [64].

We suggest that cystatins and stefins have been acquired and co-opted by a few bacterial organisms (pathogenic or comensal), and later disseminated by HGT to a few ecologically closely located but taxonomically unrelated bacteria. Bacterial stefins and cystatins therefore most probably function, like the protease inhibitors in eukaryotic pathogenic organisms, in the pathogen-host arms race [51]. The bacterial cystatins and stefins could play an important role in self defence or attack against host inflammatory and immune responses, by inhibiting cysteine cathepsins that are essential for host innate and acquired immunity. Diverse species of bacteria (either free living, symbionts or pathogens) may therefore modulate host protective responses through inhibition of cathepsins involved in antigen processing and presentation [10]. The role of cystatins as immunomodulatory proteins or as an important part of the innate immunity has been demonstrated in pathogenic nematodes [67]. Similarly, by HGT, some bacteria have evolved independently a novel anti-immune strategy to overcome host innate immunity.

While there is strong evidence that proteases are essential virulence factors for prokaryotic and eukaryotic parasites and pathogens during all stages of infection processes [68,69], there are a very few cases where protease inhibitors have been shown to assist pathogens in invading the eukaryotic hosts by inhibiting host proteases [51,70]. Stefins and cystatins with inhibitory spectra for diverse eukaryotic C1 and C13 families of cysteine proteases [2,3] are especially suited to inhibit the numerous eukaryotic host cysteine proteases during infection. In this way, the bacterial stefins and cystatins could function in the invasion and dissemination of the pathogens. One of the major roles of HGT acquired cystatins and stefins in bacteria could be to evade host immunity (in the pathogenic bacteria of animals and plants) or to protect them when in close contact with diverse eukaryotic hosts. The situation could be similar to that documented in plants, where cystatins are used in the defence against diverse viral, microbial or eukaryotic pathogens and parasites [34,50,71]. Previous studies have not considered bacterial pathogens as employing cathepsin inhibition to evade host defences, since no prokaryotic cystatins and stefins were known. We suggest that some bacteria evolved the mechanism to impair activation of the host immune response by inhibiting certain cysteine cathepsins. Inhibition of antigen processing and presentation could account for bacterial survival in the host, therefore the bacterial cystatins and stefins may constitute emergency inhibitors that defend the bacteria in acute cases of increased proteolysis [10,72,73].

## Conclusion

A comprehensive survey of the cystatin superfamily, using the extensive genomic, proteomic and transcriptomic data for Archaea, Bacteria and Eukaryota, has provided new insights into their origin, evolution and classification. Only two ancestral lineages, the stefins and the cystatins, exist in bacterial and eukaryotic genomes. In addition, 20 vertebrate-specific and three angiosperm-specific orthologous families have been discovered. Bacterial cystatins and stefins may be emergency inhibitors that enable survival of bacteria in the host, defending them from the host's proteolysis. We expect that this study will stimulate targeted functional and structural studies of the members of the cystatin superfamily present in the particular orthologous families in vertebrates and angiosperms, and especially in diverse unicellular eukaryotes and bacteria.

## Abbreviations

By: billion years; Bya: billion years ago; My: million years; Mya: million years ago; HGT: horizontal gene transfer; HRG: histidine-rich glycoprotein; LCA: last common ancestor; LUCA: last universal common ancestor.

## Authors' contributions

DK and VT conceived the design of the study. DK collected the sequence data and performed the bioinformatic, evolutionary and phylogenomic analyses. Both authors wrote the manuscript, read and approved the final version of the manuscript.

## Supplementary Material

Additional file 1 **Supplementary Table 1**. A list of eukaryotic representatives of the cystatin superfamily. Click here for file

Additional file 2 **Supplementary Table 2**. A short list of representatives of cystatins C, E/M and F from vertebrates, CRP1 from rodents and vertebrate stefins (except the amniote stefins A and B). Click here for file

Additional file 3 **Supplementary Figure 1**. **Alignment of stefins and cystatins from the unicellular eukaryotes**. The following protein sequences were used: *Giardia lamblia *(EAA37282) cystatin; *Karlodinium micrum *stefin (EC157232, Alveolata; Dinophyceae); *Euglena gracilis *stefin (EC675023); *Bigelowiella natans *stefin (DR038546, Rhizaria); *Isochrysis galbana *stefin (EC143415, Haptophyta); *Euplotes vannus *stefin (CAH04421, Chromalveolata; Ciliophora); *Capsaspora owczarzaki *stefin (EC736635, Ichthyosporea); 1Naegleria stefin (sc_81); *Monosiga brevicollis *stefin (estExt_fgenesh2_kg.C_20002 [Monbr1:35345]); 2Naegleria cystatin, (estExt_fgeneshNG_pg.C_180157 [Naegr1:79400]); *Phytophthora infestans *cystatin EPC2B (AAY21183); *Trichomonas vaginalis *cystatin (XP_001323421); *Prototheca wickerhamii *cystatin (EC178142, Chlorophyta); *Cyanophora paradoxa *cystatin (EG944090, Glaucophyta); Chlamydomonas cystatins estExt_fgenesh2_kg.C_150044 [Chlre3:183419]; *Malawimonas californiana *cystatin (EC715563); *Reclinomonas americana *cystatin (EC798377); 1 *Homo sapiens *stefin B (NP_000091) and 2 *Homo sapiens *cystatin C (CAA36497). Highly conserved QXVXG region is in bold. Click here for file

Additional file 4 **Supplementary Table 3**. Distribution of the cystatin superfamily in the plant kingdom. Click here for file

Additional file 5 **Supplementary Table 4**. Distribution of the cystatin superfamily in Holozoa (Metazoa plus Choanozoa). Click here for file

Additional file 6 **Supplementary Table 5**. Loss of the cystatin superfamily representatives in Eukaryota. Click here for file

Additional file 7 **Supplementary Figure 2**. **Cystatin from Giardia resembles the most ancestral eukaryotic cystatin**. The following protein sequences were used: *Giardia lamblia *(EAA37282) cystatin; *Euglena gracilis *stefin (EC675023); Naegleria cystatin, (estExt_fgeneshNG_pg.C_180157 [Naegr1:79400]); *Phytophthora infestans *cystatin EPC2B (AAY21183); *Trichomonas vaginalis *cystatin (XP_001323421); *Reclinomonas americana *cystatin (EC798377); and *Homo sapiens *cystatin C (CAA36497). Highly conserved QXVXG region is in bold. Click here for file

Additional file 8 **Supplementary Figure 3**. **Legumain binding motif in bifunctional cystatins is conserved in all land plants and in some green algae**. The following protein sequences were used: Physcomitrella (estExt_gwp_gw1.C_2380025 [Phypa1_1:195387]); Oryza Oryzacystatin-12 (NP_001042702); Marchantia (BJ841987); Selaginella (BM402705); *Zamia fischeri *(DY036558); *Pseudotsuga menziesii *(CN639199); *Ginkgo biloba *(EX934790); *Ceratopteris richardii *(BE641752); *Amborella trichopoda *(CK755139) and green alga *Scenedesmus obliquus *cystatin (EC184546 + EC184713). Highly conserved QXVXG region and legumain binding motif (SNSL) are in bold. Click here for file

Additional file 9 **Supplemetary Figure 4**. **Gain of signal peptide in some eukaryotic stefins**. The following protein sequences were used: *Karlodinium micrum *stefin (EC157232, Alveolata; Dinophyceae); *Capsaspora owczarzaki *stefin (EC736635, Ichthyosporea); *Hyperamoeba dachnaya *stefin (EC853881), *Nannochloropsis oculata *stefin (EE109499, stramenopiles; Eustigmatophyceae); *Euglena gracilis *stefin (EC675023); Monosiga stefin (estExt_fgenesh2_kg.C_20002 [Monbr1:35345]); *Dictyostelium discoideum *(XP_629960) stefin; Reclinomonas stefin (EC788759, Jakobidae); and *Homo sapiens *stefin B (NP_000091). Highly conserved G and QXVXG region are in bold. The names of the taxa where stefins gained signal peptide are in bold. Click here for file

Additional file 10 **Supplementary Table 6**. Ancestral states for the cystatin superfamily in eukaryotic supergroups. Click here for file

Additional file 11 **Supplementary Figure 5**. **Functionally important structural motif of eukaryotic cystatins is conserved in the bacterial stefins and cystatins**. Highly conserved QXVXG region is in bold. Eukaryotic cystatin (Giardia) and stefin (Euglena) have been included. Click here for file
