# Supplementary material for: Phylogenomic analysis of the cystatin superfamily in eukaryotes and prokaryotes
Source: BMC Evol Biol. 2009 Nov 18;9:266. doi: 10.1186/1471-2148-9-266 (PMC2784779; doi:10.1186/1471-2148-9-266)
Supplement: Additional file 1 — Supplementary Table 1. A list of eukaryotic representatives of the cystatin superfamily. [file 1471-2148-9-266-S1.PDF]

Supplementary Table 1. A list of eukaryotic representatives of the cystatin superfamily.

Taxonomic group	species name	stefins	cystatins	multicystatins
Diplomonadida	Giardia lamblia	-	EAA37282	-
Parabasalia	Trichomonas vaginalis	-	EAY22465, AAHC01000026, XP_001323421, EAY03179	EAY15373, CV219339, CO576890,
	Tritrichomonas foetus		CX154995, CX158473, CX156087	CX154814
Oxymonadida	Streblomastix strix		EC194219, EC191702, EC192438, EC190685	EC193035
Euglenozoa	Euglena gracilis	EC675023		
	Euglena longa	EC630014		
Heterolobosea	Naegleria gruberi	e_gw1.2.345.1 [Naegr1:29629], sc_81	fgeneshNG_pg.scaffold_8000191 [Naegr1:63954], fgeneshNG_pg.C_180157 [Naegr1:79400]	fgeneshHS_pg.scaffold_3000182 [Naegr1:45778]
	Sawyeria marylandensis		EC821201, EC820369, EC823730	
Jakobidae	Reclinomonas americana	EC788759	EC797076, EC798377	
	Jakoba libera		EC691959	
	Jakoba bahamiensis		EC685686	
	Histiona aroides		EC850908	
Malawimonas	Malawimonas jakobiformis	EC722292	EC719335	
	Malawimonas californiana	EC716638	EC716164, EC715563	
ciliates	Euplotes vannus	CAH04421		

	Isotricha sp.	CF182047	CF181950, AM054335	
	Tetrahymena thermophila	EV837790	EAR88621, XP_001008866	XP_001023354, XP_001023352, XP_001012461,
	Epidinium ecaudatum	AM053275, AM051603	AM053709	
	Eudiplodinium maggii	AM053836	AM054178	
	Entodinium caudatum	AM051580	AM052756	
	Paramecium tetraurelia	XP_001431119, XP_001458463, XP_001425357, XP_001451197	XP_001432545, XP_001458462, XP_001460294, XP_001433933, XP_001426146, XP_001437546, XP_001447637, XP_001451867, XP_001462530, XP_001449533,	XP_001456057, XP_001440218, XP_001454624, XP_001440217,
	Polyplastron multivesiculatum		AM055362	
Dinophyta	Amphidinium carterae	CF066094		
	Karlodinium micrum	EC157232		
Perkinsea	Perkinsus marinus	AAXJ01000159		
diatoms	Phaeodactylum tricornutum	-	CT871415	
	Thalassiosira pseudonana	-	fgenesh1_pg.C_chr_19a_19000070	
Blastocystis	Blastocystis hominis	EC651417		
Oomycetes	Phytophthora		AAY21184, AATU01003822, AAY21183,	

	infestans		AAY21185,
	Phytophthora sojae		estExt_fgenesh1_pg.C_500126 [Physo1_1:137090], estExt_fgenesh1_pg.C_1040055 [Physo1_1:141871], jgi|Physo1_1|138161|estExt_fgenesh1_pg.C_600120, jgi|Physo1_1|138157|estExt_fgenesh1_pg.C_600116
	Phytophthora ramorum		fgenesh1_pg.C_scaffold_3000260 [Phyra1_1:73503], jgi|Phyra1_1|75664|fgenesh1_pg.C_scaffold_14000026, jgi|Phyra1_1|94233|C_scaffold_14000006, jgi|Phyra1_1|86235|fgenesh1_pg.C_scaffold_553000003
	Phytophthora brassicae		ES290097
	Aphanomyces cochlioides		ES277215
	Plasmopara halstedii		CB174713
	Saprolegnia parasitica		DN616890
Eustigmatophyceae	Nannochloropsis oculata	EE109499	
Labyrinthulida	Schizochytrium sp.	EH403078	
Rhizaria	Bigelowiella natans	DR038546	
	Cercomonas longicauda	EW704828, EW705012	
Haptophyta	Isochrysis galbana	EC143415	
	Emiliania huxleyi	EG033655	
	Pavlova lutheri		EC176020
Cryptophyta	Guillardia theta	AW342576	

Glaucophyta	Cyanophora paradoxa		EG944090
green algae	Helicosporidium sp.		CX129376
	Scenedesmus obliquus		EC184546 + EC184713
	Prototheca wickerhamii		EC178142
	Chlamydomonas reinhardtii		BQ822357
	Volvox carteri		FD886153
land plants			
Amoebozoa	Dictyostelium discoideum	XP_629960, XP_642845, EAL67198	
	Hyperamoeba dachnaya	EC853881	
	Polysphondylium pallidum	EC762009	
	Acanthamoeba castellanii	EC099592	
	Physarum polycephalum	EL577982	
Choanozoa	Capsaspora owczarzaki	EC736635	EC736599
	Monosiga brevicollis	XP_001742194	-
	Monosiga ovata		DC515438, DC491487, DC515437
**Metazoa**			
Porifera	Amphimedon queenslandica	gnl|ti|1458392754	
	Oscarella		EC376733

	carmela			
Cnidaria	Hydra magnipapillata	DR435266	CN566221, DN244254, BP506435, DN240932	
	Nematostella vectensis	DV091807	XP_001634815,	
Ctenophora	Mnemiopsis leidyi		FC463031	
	Pleurobrachia pileus	CU418341		
Acoela	Convoluta pulchra	EV601159		
**Protostomia**				
Ecdysozoa				
Arthropoda	Carcinus maenas	DY656536		
	Manduca sexta			BAE97580
	Rhipicephalus microplus		CK173766	
Onychophora	Epiperipatus sp.	AM500237		
Nematoda	Trichinella spiralis	ES566455	BQ693291	
Tardigrada	Hypsibius dujardini	CO508333		
Lophotrochozoa				
Platyhelminthes	Fasciola hepatica			AJ312374
	Schistosoma japonicum	AY915621	ABL86193	
Mollusca	Biomphalaria glabrata	CK989072	EB709538	
	Euprymna scolopes			DW257290
Annelida	Helobdella robusta	e_gw1.8.139.1 [Helro1:70492]	EY322967	

Rotifera	Brachionus plicatilis	BJ979696		
**Deuterostomia**				
Echinodermata	Strongylocentrotus purpuratus	XP_784732	-	
Xenoturbellidae	Xenoturbella bocki	-	AM159914, EC906440	
**Chordata**				
-Cephalochordata	Branchiostoma floridae	BW800420	e_gw.68.153.1 [Brafl1:216874]	BW701309 + BW720095
-Urochordata	Halocynthia roretzi	DB624191	-	
-Vertebrata	Petromyzon marinus	EG021530	EE738468	EB081161
