# Supplementary material for: Phylogenomic analysis of the cystatin superfamily in eukaryotes and prokaryotes
Source: BMC Evol Biol. 2009 Nov 18;9:266. doi: 10.1186/1471-2148-9-266 (PMC2784779; doi:10.1186/1471-2148-9-266)
Supplement: Additional file 2 — Supplementary Table 2. A short list of representatives of cystatins C, E/M and F from vertebrates, CRP1 from rodents and vertebrate stefins (except the amniote stefins A and B). [file 1471-2148-9-266-S2.PDF]

Supplementary Table 2. A short list of representatives of cystatins C, E/M and F from vertebrates, CRP 1 from rodents and vertebrate stefins (except the amniote stefins A and B).

Taxonomic group	Species name	proto-cystatin	cystatin F	cystatin C	cystatin E/M	CRP-1	stefin
Cyclostomata	Eptatretus burgeri	BJ665000					BJ653099
	Petromyzon marinus	EE738468	EE738468				EG021530
Chondrichthyes	Leucoraja erinacea	DT046118			EE992410		EE988223
	Squalus acanthias		EG361298				EE627959
Actinopterygii	Acipenser transmontanus			DR975381			DR975179
	Oncorhynchus mykiss		BX876069	CU063610			CX718739
Amphibia	Xenopus tropicalis		AAH88052	DC184348			EL852078
	Ambystoma mexicanum			CN051834			CN051554
»Reptilia«	Anolis carolinensis		GENSCAN00000063542	scaffold_425.13	scaffold_834.9		
Aves	Gallus gallus		XP_415013	BX257695			
Prototheria	Ornithorhynchus anatinus		XP_001511481	EE678047	XP_001379474		
Metatheria	Monodelphis domestica		XP_001382090	XP_001382093	ENSOANP00000003006		
Eutheria	Mus musculus		BAE41893	AF311741	AAH61036	XP_923268, XP_485073, XP_001481123	
	Rattus norvegicus		XP_001056457	NP_036969	NP_598250	NP_036850, XP_001057954, XP_575250, AAA42345, CAA78385,	
