# Supplementary material for: Phylogenomic analysis of the cystatin superfamily in eukaryotes and prokaryotes
Source: BMC Evol Biol. 2009 Nov 18;9:266. doi: 10.1186/1471-2148-9-266 (PMC2784779; doi:10.1186/1471-2148-9-266)
Supplement: Additional file 3 — Supplementary Figure 1. Alignment of stefins and cystatins from the unicellular eukaryotes. The following protein sequences were used: Giardia lamblia (EAA37282) cystatin; Karlodinium micrum stefin (EC157232, Alveolata; Dinophyceae); Euglena gracilis stefin (EC675023); Bigelowiella natans stefin (DR038546, Rhizaria); Isochrysis galbana stefin (EC143415, Haptophyta); Euplotes vannus stefin (CAH04421, Chromalveolata; Ciliophora); Capsaspora owczarzaki stefin (EC736635, Ichthyosporea); 1Naegleria stefin (sc_81); Monosiga brevicollis stefin (estExt_fgenesh2_kg.C_20002 [Monbr1:35345]); 2Naegleria cystatin, (estExt_fgeneshNG_pg.C_180157 [Naegr1:79400]); Phytophthora infestans cystatin EPC2B (AAY21183); Trichomonas vaginalis cystatin (XP_001323421); Prototheca wickerhamii cystatin (EC178142, Chlorophyta); Cyanophora paradoxa cystatin (EG944090, Glaucophyta); Chlamydomonas cystatins estExt_fgenesh2_kg.C_150044 [Chlre3:183419]; Malawimonas californiana cystatin (EC715563); Reclinomonas americana cystatin (EC798377); 1 Homo sapiens stefin B (NP_000091) and 2 Homo sapiens cystatin C (CAA36497). Highly conserved QXVXG region is in bold. [file 1471-2148-9-266-S3.PDF]

```
Karlodinium        --------------MPRNGKALKPEDDTVTTEPVLVGGHSIEKEMDEDVKAIVLSLKARM 46
Euplotes           -----------------------------------MGAYG-ELKTDAEGLAKVAGWKSHV 24
Capsaspora         -----MAALVPNSARGLVPNSARGNKPKQSVMSGMPGGTSNARDADAETQSILDQVKSQA 55
Monosiga           -------------------------------MA-MVGGFGAPRDADEEIQQVADAVKSDV 28
1Homo              ---------------------------------MMCGAPSATQPATAETQHIADQVRSQL 27
Euglena            ---------------------------------MLCGGAGAEQPANDEIRQLCLTVKDGV 27
Bigelowiella       ---------------------------------MMTGGYTDFRKAEEKEIEIVKKMAEDI 27
1Naegleria         --------------------------------MIEVGQHNKHHTPTMEASQMAFIVKPMV 28
Prototheca         -MSKYVAFMVLALCALATASPLISPLEELCGAEGLIGGYSKANISDPTSQEVFEAVADEF 59
Chlamydomonas      -------------MAKRVLIALAAFVMLNAATATIVGGSSKAAVSDPDVVHAANFVVSSA 47
Trichomonas        --------------------------MSCCGGRCGCGGVKPANVDDEHVIQAFKDAVALA 34
Malawimonas        --------------MKTAFVLILAVTLGVAVSAIMTGAPNKMDVSDPRVQELASFAMNRI 46
Cyanophora         ----MNKAASLTFLLLALFSVHVALAKMSPPGAGLPGGLFPASAKDEGVTEAAEFAVQEI 56
2Homo              MAGPLRAPLLLLAILAVALAVSPAAGSSPGKPPRLVGGPMDASVEEEGVRRALDFAVGEY 60
Reclinomonas       ------MRATLLLLVLLIAATVLVALCAEAARMPMPGGQFPVGSGDLEREDIVAAAQAAV 54
Phytophthora       -------------MSFLRPTLALLAVTALVTTSGQLNGYSKKEVTPEDTELLQKAQSNVS 47
Isochrysis         ----------------------------------MVGGTSAMDISDERLQSAVQAVVPQF 26
Giardia            ---------------------------------MLAGGWTELAPADVN-SKVREAAAAKI 26
2Naegleria         --------MFQRSSTAILLLLIACLMMIGFTSAVIPGGFSHNKKPSAKRIAKFTSFLSSK 52
                                                       .

Karlodinium        EEKLAKT----------FTTFEPTRYTSQIVAGTVYQVKIKC-------DDEFIQAKIVK 89
Euplotes           EATAGTT----------FDAFEVIHYKTQIVAGTNWEAKVKIG------DDEYAHVVIHE 68
Capsaspora         EEKAGKS----------FSQFVAKQVATQVVAGTNFFVKADIG------NGEQVHVRIFR 99
Monosiga           VAKIGKD----------VEQFKAIQVSTQVVAGTNYLIKVDVG------SNEFVHIKVFR 72
1Homo              EEKENKK----------FPVFKAVSFKSQVVAGTNYFIKVHVG------DEDFVHLRVFQ 71
Euglena            HAAARNTGFA-----GDFTKYEPVSYKTQVVAGTNFFIKLAVA------EDQFLHARIFK 76
Bigelowiella       AKKIESEA---------FKSFKVTGVKTQVVAGTNYTFQVEA-------DEHKLHVTIWR 71
1Naegleria         EKQTSMT----------YKNFELIEHTTQVVNGILHKMKIKVG------DEEYIHLKVIE 72
Prototheca         ITANNATIT------CDDFVVEPLQACSQVVAGTNFEVLLHITCP---ATDESVTVYGTA 110
Chlamydomonas      NTNACSGLCAGLQKEGELKLVKVLSASTQVVAGVNVHLELLMADD---TGKQTVVTSTVW 104
Trichomonas        NQKNGTN----------LEFVELITATQQVVSGFIFEGVVKTN------DGDYKAK--IW 76
Malawimonas        RAASNSVET--------TQMLRVVSAESQVVAGVKYSLVLEVQTG---SQAPSQHQIVIV 95
Cyanophora         SNRSNSMKS--------LDLVQVLEVQRQVVAGLNFYLKLSVT-----TGDKNNQIYNAV 103
2Homo              NKASNDMYH--------SRALQVVRARKQIVAGVNYFLDVELGRTTCTKTQPNLDNCPFH 112
Reclinomonas       SHISSTQNLR-------VQLSRVTRAATQVVAGLNYYLTVELDELG--GAGNAKRTYDVV 105
Phytophthora       AYNSDVTSR--------ICYLKVDSLETQVVSGENYKFHVSGCS-----VNSDKELGGCA 94
Isochrysis         QAMAQGQGRN-----GMVSQLKIVEAQAQVVAGINYFVKVHIG------HDEYAHMRIYD 75
Giardia            AESVSGA-----------TIAEVIKASSQVVRGVNTMLLTRLS------TGAHYIVVVWF 69
2Naegleria         LAAKYPT------------ITQIIDIQQQVVAGVMYKVTALAIDSN--GQEKTIKATIFE 98
                                               *:* *

Karlodinium        PLPHAGSPPDLMECVGGKSESDAFSF-------- 115
Euplotes           KLPHTGEEPSCSSFNGGKTLDDPLGS-------- 94
Capsaspora         SLPP-AQALSVHSIQTGKTASDPLVHF------- 125
Monosiga           SLPP-FQH-ELKAVETGKGATDALSNIE------ 98
1Homo              SLPHENKPLTLSNYQTNKAKHDELTYF------- 98
Euglena            PLPCNGANPEVHSVQINKALADPVEHF------- 103
Bigelowiella       KLDQTTELTKVVKV-------------------- 85
1Naegleria         -NPQ--GGLTVNEISPGKKMNDML---------- 93
Prototheca         FVPLPSSGTQTVTVDEVEQVPE------------ 132
Chlamydomonas      SRPWLASKNDAAQPATQITALTFKPLDGTLE--- 135
Trichomonas        CKPG-NTEKELQSFEKY----------------- 92
Malawimonas        ERPWENHKEIVSHQLV------------------ 111
Cyanophora         VYRNLQGQFSLTSFSPA----------------- 120
2Homo              DQPHLKRKAFCSFQIYAVPWQGTMTLSKSTCQDA 146
Reclinomonas       VYRGFDRKYSVSRVSEVHETSFGSE--------- 130
Phytophthora       NQNCESSKYDIVIYSQSWTNTLKVTSITPAN--- 125
Isochrysis         HFGN----IELTDIQLSKTKTDRLEYF------- 98
Giardia            DLKNYIVTTLKEYTGNLANFTWPMRE-------- 95
2Naegleria         PLPHAIQAGQSKLQLKDVKEL------------- 119
```

Supplementary Figure 1
