# Supplementary material for: Phylogenomic analysis of the cystatin superfamily in eukaryotes and prokaryotes
Source: BMC Evol Biol. 2009 Nov 18;9:266. doi: 10.1186/1471-2148-9-266 (PMC2784779; doi:10.1186/1471-2148-9-266)
Supplement: Additional file 4 — Supplementary Table 3. Distribution of the cystatin superfamily in the plant kingdom. [file 1471-2148-9-266-S4.PDF]

Supplementary Table 3. Distribution of the cystatin superfamily in the plant kingdom.

Taxonomic group	group A cystatins	group B cystatins	group C cystatins	ancestral cystatins	multicystatins	bifunctional cystatins
Glaucophyta	□	□	□	■	□	□
Rhodophyta	□	□	□	□	□	□
Chlorophyta	■	□	□	■	□	■
Embryophyta	■	□	□	□	□	■
Marchantiophyta	■	□	□	□	□	■
Bryophyta	■	□	□	□	□	■
Lycopodiophyta	■	□	□	□	□	■
Pteridophyta	■	□	□	□	□	■
Gymnosperms	■	□	□	□	□	■
Angiosperms	■	■	■	□	■	■
-basal angiosperms	■	■	■	□	□	■
-magnoliids	■	■	■	□	□	■
-monocots	■	■	■	□	□	■
-eudicots	■	■	■	□	■	■

Presence is marked with the black square and the absence with the white square.
