# Supplementary material for: Phylogenomic analysis of the cystatin superfamily in eukaryotes and prokaryotes
Source: BMC Evol Biol. 2009 Nov 18;9:266. doi: 10.1186/1471-2148-9-266 (PMC2784779; doi:10.1186/1471-2148-9-266)
Supplement: Additional file 5 — Supplementary Table 4. Distribution of the cystatin superfamily in Holozoa (Metazoa plus Choanozoa). [file 1471-2148-9-266-S5.PDF]

Supplementary Table 4. Distribution of the cystatin superfamily in Holozoa (Metazoa plus Choanozoa).

Taxonomic group	stefins	cystatins	multicystatins
**Choanozoa**	■	■	□
**Metazoa**	■	■	■
Porifera	■	■	□
Cnidaria	■	■	□
Ctenophora	■	■	□
Placozoa	□	□	□
Acoela	■	□	□
**Protostomia**	■	■	■
Ecdysozoa	■	■	■
Arthropoda	■	■	■
Nematoda	■	■	□
Tardigrada	■	□	□
Lophotrochozoa	■	■	■
Platyhelminthes	■	■	■
Mollusca	■	■	■
Annelida	■	■	□
Rotifera	■	□	□
**Deuterostomia**	■	■	■
Echinodermata	■	□	□
Xenoturbellidae	□	■	□
Hemichordata	□	□	□
**Chordata**	■	■	■
-Cephalochordata	■	■	■
-Urochordata	■	□	□
-Vertebrata	■	■	■

Presence is marked with the black square and the absence with the white square.
