# Supplementary material for: Phylogenomic analysis of the cystatin superfamily in eukaryotes and prokaryotes
Source: BMC Evol Biol. 2009 Nov 18;9:266. doi: 10.1186/1471-2148-9-266 (PMC2784779; doi:10.1186/1471-2148-9-266)
Supplement: Additional file 6 — Supplementary Table 5. Loss of the cystatin superfamily representatives in Eukaryota. [file 1471-2148-9-266-S6.PDF]

Supplementary Table 5. Loss of the cystatin superfamily representatives in Eukaryota.

Taxonomic group	complete loss	stefins	cystatins
diplomonads		?■	
parabasalians		?■	
oxymonads		?■	
Kinetoplastida	■		
Apicomplexa	■		
Perkinsus			■
diatoms		■	
Oomycetes		■	
Rhizaria			■
Glaucophyta		?■	
red algae	■		
green algae		■	
land plants		■	
Amoebozoa			■
Fungi	■		
Placozoa	■		
insects		■	
Nematoda		■	
echinoderms			■
urochordates			■

Loss is marked with the black square.
