# Supplementary material for: Phylogenomic analysis of the cystatin superfamily in eukaryotes and prokaryotes
Source: BMC Evol Biol. 2009 Nov 18;9:266. doi: 10.1186/1471-2148-9-266 (PMC2784779; doi:10.1186/1471-2148-9-266)
Supplement: Additional file 7 — Supplementary Figure 2. Cystatin from Giardia resembles the most ancestral eukaryotic cystatin. The following protein sequences were used: Giardia lamblia (EAA37282) cystatin; Euglena gracilis stefin (EC675023); Naegleria cystatin, (estExt_fgeneshNG_pg.C_180157 [Naegr1:79400]); Phytophthora infestans cystatin EPC2B (AAY21183); Trichomonas vaginalis cystatin (XP_001323421); Reclinomonas americana cystatin (EC798377); and Homo sapiens cystatin C (CAA36497). Highly conserved QXVXG region is in bold. [file 1471-2148-9-266-S7.PDF]

```
Homo              MAGPLRAPLLLLAILAVALAVSPAAGSSPGKPPRLVGG--PMDAS-VEEEGVRRALDFAV 57
Reclinomonas      ----MRATLLLLVLLIAATVLVALCAEAARMP--MPGGQFPVGSGDLEREDIVAAAQAAV 54
Phytophthora      -MSFLRPTLALLAVTALVTTSGQLNGYSKKEVT-------------PEDTELLQKAQSNV 46
Trichomonas       -----------------MSCCGGRCGCGGVKP------------ANVDDEHVIQAFKDAV 31
Euglena           ------------------MLCG---GAGAEQP------------ANDEIRQLCLTVKDGV 27
Giardia           ------------------MLAG---GWTELAP--------------ADVNSKVR--EAAA 23
Naegleria         -MFQRSSTAILLLLIACLMMIG---FTSAVIPG-----------GFSHNKKPSAKRIAKF 45
                                                                 .

Homo              GEYNKASNDMYHSRALQVVRARKQIVAGVNYFLDVELGR-TTCTKTQPNLDNCPFHDQPH 116
Reclinomonas      SHISSTQN--LRVQLSRVTRAATQVVAGLNYYLTVELDE-LGGAGNAKRTYDVVVYRGFD 111
Phytophthora      SAYNSDVT--SRICYLKVDSLETQVVSGENYKFHVSG----CSVNSDKELGGCANQNCES 100
Trichomonas       ALANQKNG--TNLEFVELITATQQVVSG--FIF-------EGVVKTNDGDYKAKIWCKPG 80
Euglena           HAAARNTGFAGDFTKYEPVSYKTQVVAGTNFFIKLAVAE-DQFLHAR--IFKPLPCNGAN 84
Giardia           AKIAESVS---GATIAEVIKASSQVVRGVNTMLLTRLS--TGAHYIVVVWFDLKNYIVTT 78
Naegleria         TSFLSSKLAAKYPTITQIIDIQQQVVAGVMYKVTALAIDSNGQEKTIKATIFEPLPHAIQ 105
                                  .      *:* *    .

Homo              LKRKAFCSFQIYAVPWQGTMTLSKSTCQDA 146
Reclinomonas      RKYSVSRVSEVHETSFGSE----------- 130
Phytophthora      SKYDIVIYSQSWTNTLKVTSITPAN----- 125
Trichomonas       NTEKELQSFEKY------------------ 92
Euglena           PEVHSVQINKALADPVEHF----------- 103
Giardia           LKEYTGNLAN-FTWPMRE------------ 95
Naegleria         AGQSKLQLKDVKEL---------------- 119
                           .
```

Supplementary Figure 2
