# Supplementary material for: Phylogenomic analysis of the cystatin superfamily in eukaryotes and prokaryotes
Source: BMC Evol Biol. 2009 Nov 18;9:266. doi: 10.1186/1471-2148-9-266 (PMC2784779; doi:10.1186/1471-2148-9-266)
Supplement: Additional file 8 — Supplementary Figure 3. Legumain binding motif in bifunctional cystatins is conserved in all land plants and in some green algae. The following protein sequences were used: Physcomitrella (estExt_gwp_gw1.C_2380025 [Phypa1_1:195387]); Oryza Oryzacystatin-12 (NP_001042702); Marchantia (BJ841987); Selaginella (BM402705); Zamia fischeri (DY036558); Pseudotsuga menziesii (CN639199); Ginkgo biloba (EX934790); Ceratopteris richardii (BE641752); Amborella trichopoda (CK755139) and green alga Scenedesmus obliquus cystatin (EC184546 + EC184713). Highly conserved QXVXG region and legumain binding motif (SNSL) are in bold. [file 1471-2148-9-266-S8.PDF]

```
Physcomitrella      ---------------------------------------------------MLSGGKQEV 9
Ceratopteris        ------------------------------------------------------------
Pseudotsuga         -----------KVTIAIT-----------IALLCFCG------TSTLAFY---IGAIRDV 29
Ginkgo              ---------GERVWFSIRGITTGLLFIIFSATLCMCA------MAASTFL---AGGIKDV 42
Zamia               -------MQGSRYWGKPGGTG---WMPLFLVILILLG------DTGLFVMG-KLGGRRDI 43
Amborella           -----------MRSIAFS-----------VVVVILLQ------SLGAMST---LGGLKDV 29
Oryza               MRVAATTRPASSSAAAPLPLFLLLAVAAAAAALFLVG------SASLAMAGHVLGGAHDA 54
Marchantia          ---MNTRKQQQQHFVQRALWTRVILFVLVVGAVCYVGASLCDAKESGADMAGLMGAPKDV 57
Selaginella         ----------------------LLGFLVIALFLCCS------------AMSKLVGAPKEV 26
Scenedesmus         ------------------------------------------------------------

Physcomitrella      DLQNSNNLEIDEAAKFAVAEHNDRENS---LEKLTFSKVVSCHMQVVAGSMYYLVIEVEE 66
Ceratopteris        ------------------------------------------------------------
Pseudotsuga         PN-FQHSPLINRLAGFALNEFNKQQGAQ--NAHLSLSKVVRAREQMVSGMVYYLTMEALD 86
Ginkgo              PN-FQHDPEIQSLARFAVDEYNKQQ-----NILLSFSKVVKAQQQVVAGMIYYLTIEAVD 96
Zamia               PG-FQNSIEIQDLARFAVEQYNKQQ-----NTLLSFSRVVKAQEQVVAGTLYYLTIEAKD 97
Amborella           TGSNQNSAEIEELARFAVQEHNKKS-----NSLLEFARVVKAKEQVVAGTLYHLTLEAID 84
Oryza               PS-AANSVETDALARFAVDEHNKRE-----NALLEFVRVVEAKEQVVAGTLHHLTLEALE 108
Marchantia          SN-AENSVEMDDMGRFAVDQYNTKE-----GKNLSFKNVVSAKKQVVSGTMYHMVIEAHG 111
Selaginella         KE-DENSVEIQDHAKFAVDEHNRQNP----NANLSFKKLVSAKKQVVAGSLYHLTIEAES 81
Scenedesmus         ----PIDARTNEIATFAVEQLKHGKANFPIKGNLQLAKVVSAKRQVVSGTNHILVVEAAD 56
                                                                   :*

Physcomitrella      -GSSIKLYEAKVWVKPWQNFKKLEEFKLKDAGV----TSADLGVRTGGPHSTGRGISAPP 121
Ceratopteris        ---RINIYEAKVHIKPWERFKSLVHFGKAESSF----TTADLGAHIG-DGSNHMGLRVVP 55
Pseudotsuga         SHRTTGSYLAQVWVVPWRNFTQLEEFKQLDADK----------GKLDGAQAPLG--DAVG 134
Ginkgo              GHESHR-YSAKILVVEWRNIKQLQEFKPMPPQQPSSLTPADLGGKQGGSRESGW--QTVP 153
Zamia               -HGTPRLYEAKIWVKPWQNFKQLQEFKPVSHRP---FTAADLGVKQEGLLGTGW--RTVS 151
Amborella           -AGKNKLYEAKVWLKPWENFKELKEFNHVGDSS---LTSSDLGAKRDN-HGPGW--REVP 137
Oryza               -AGRKKVYEAKVWVKPWLDFKELQEFRNTGDAT--TFTNADLGAKKGG-HEPGW--RDVP 162
Marchantia          -DDSVKMYDAKVWHKPWENHKSLEHFKPQ--ES----------SANDSPNTEVAGMRSVP 158
Selaginella         -PEGSKLYNAQVWTKEWEGSKQLKEFKPH--ST----------EDN-------TGVRKIP 121
Scenedesmus         -DAGPKTLEVTVWEKLSCNVKENDAAMELTHFK-------------------LVGPAAEA 96
                             . :        ..

Physcomitrella      SGKQSWPTDDLVVQEAAEHAMKMLQQGSNSLASYELSEIVSADAELSDESADFELLLKIK 181
Ceratopteris        S-------EDPAVKEAAKEALKHIQARSNSLIPYELKEVMQAHAEVNDEHTKFHILLNVL 108
Pseudotsuga         A-------QDPIVKEAAENAVKIIQQRSNSLMPYKLQEIVSAKEKVVNALKIFNLLLKIK 187
Ginkgo              T-------QDPVVQEAAENAVKIIQQRSNSLAPYQLQDVLSA------------------ 188
Zamia               V-------HDPVIQEAAELAVKNIEQKSNSLASYELQEILLAKAEVIEDWDR-NLIC--- 200
Amborella           A-------QDPVVQEAAAHAVKTIQQRSNSLAPYELLEILLARAEVIEESAKFDMLLKLK 190
Oryza               V-------HDPVVKDAADHAVKSIQQRSNSLFPYELLEIVRAKAEVVEDFAKFDILMKLK 215
Marchantia          V-------GDPVIKEAAEHALKGLNDRSNSLVPYELRQVMTAHAEATDEHTNFDLHIKVA 211
Selaginella         V-------DDPVVREAAEHALKGLQQRSNSLVPYELRHVVEAQAKVTKDNTDLDLLLKVK 174
Scenedesmus         G--------DDQWSKAAQQGVAQLNQRSNSLFPYQLLRVLSATP-LNDGSSNTELLVEVK 147
                             *    .**  .:  ::  **** .*:*  :: *

Physcomitrella      RGAKEEHFKSEIHRTGDGDWSVKHVTLQ--------- 209
Ceratopteris        RGPKEEQFKAELTRTVLGEWSLKDLQQHHEGAL---- 141
Pseudotsuga         WEN---------------------------------- 190
Ginkgo              -------------------------------------
Zamia               -------------------------------------
Amborella           EGKQGGKI----------------------------- 198
Oryza               RGNKEEKFKAEVHKNLEGAFVLNQMQQEHDESSSQ-- 250
Marchantia          RGAKEEEMKAELHRTADGKWSLKHAGPM--------- 239
Selaginella         RGEREEEVRAAVY------------------------ 187
Scenedesmus         RGDKQEKFALTVKPAADAHFQLVKFHQHAAEGPATS- 183
```

Supplementary Figure 3
