# Supplementary material for: Phylogenomic analysis of the cystatin superfamily in eukaryotes and prokaryotes
Source: BMC Evol Biol. 2009 Nov 18;9:266. doi: 10.1186/1471-2148-9-266 (PMC2784779; doi:10.1186/1471-2148-9-266)
Supplement: Additional file 9 — Supplemetary Figure 4. Gain of signal peptide in some eukaryotic stefins. The following protein sequences were used: Karlodinium micrum stefin (EC157232, Alveolata; Dinophyceae); Capsaspora owczarzaki stefin (EC736635, Ichthyosporea); Hyperamoeba dachnaya stefin (EC853881), Nannochloropsis oculata stefin (EE109499, stramenopiles; Eustigmatophyceae); Euglena gracilis stefin (EC675023); Monosiga stefin (estExt_fgenesh2_kg.C_20002 [Monbr1:35345]); Dictyostelium discoideum (XP_629960) stefin; Reclinomonas stefin (EC788759, Jakobidae); and Homo sapiens stefin B (NP_000091). Highly conserved G and QXVXG region are in bold. The names of the taxa where stefins gained signal peptide are in bold. [file 1471-2148-9-266-S9.PDF]

```
Euglena              ----------------------------MLCGGAG-AEQPANDEIRQLCLTVKDGVHAAA 31
Dictyostelium        ----------------------------MTLGGLKPEVHAANDEIRQVVAKVADELKSKL 32
Hyperamoeba          --------MNMKSVLLVCLVFAVAFSSAKLVGGLS-DERPVDADVISIANEVREQAQAKL 51
Reclinomonas         ---------------------------MQRAGRVS-EAKPIDDAARAAAEAVRHEAEAQA 32
Capsaspora           MAALVPNSARGLVPNSARGNKPKQSVMSGMPGGTS-NARDADAETQSILDQVKSQAEEKA 59
Monosiga             --------------------------MA-MVGGFG-APRDADEEIQQVADAVKSDVVAKI 32
Homo                 ----------------------------MMCGAPS-ATQPATAETQHIADQVRSQLEEKE 31
Karlodinium          ---------MPRNGKALKPEDDTVTTEPVLVGGHSIEKEM-DEDVKAIVLSLKARMEEKL 50
Nannochloropsis      -----------------------MSPPSGLIGGFGATKETPDVETTALLSSVRGEVEKQL 37
                                                    *      .            :

Euglena              RNTGFAGDFTKYEPVSYKTQVVAGTNFFIKLAVAEDQFLHARIFKPLPCNGANPEVHSVQ 91
Dictyostelium        -NT------TEVEPVSYKTQLVAGTNYFIKVKTPAG-FAHARVYKDLQQNHS---VHSVK 81
Hyperamoeba          PNES----FQSYTPISYSTQTVAGVNYFIRVDIGAEYHIVLRVFKSLRGELS---LHSTK 104
Reclinomonas         G-HA----FEKYEPVSYTSQVVAGTNFFIKVNTGAD-HLHLRVFRDFGGAHS---LHSLQ 83
Capsaspora           GKS-----FSQFVAKQVATQVVAGTNFFVKADIGNGEQVHVRIFRSLPP-AQALSVHSIQ 113
Monosiga             GKD-----VEQFKAIQVSTQVVAGTNYLIKVDVGSNEFVHIKVFRSLPP-FQH-ELKAVE 85
Homo                 NKK-----FPVFKAVSFKSQVVAGTNYFIKVHVGDEDFVHLRVFQSLPHENKPLTLSNYQ 86
Karlodinium          AKT-----FTTFEPTRYTSQIVAGTVYQVKIKCDD-EFIQAKIVKPLPHAGSPPDLMECV 104
Nannochloropsis      STT-----FSTFEPHSVRTQVVAGTNYRCKVHVGDSKYVHMVVHQPLPHTKEPAKLMSAE 92
                                  .    :* ***. :  :            : : :        :

Euglena              INKALADP-VEHF- 103
Dictyostelium        ADGITEESEIVYF- 94
Hyperamoeba          SNVGKNDA-LAYF- 116
Reclinomonas         AGKSADDE-IAYF- 95
Capsaspora           TGKTASDP-LVHF- 125
Monosiga             TGKGATDA-LSNIE 98
Homo                 TNKAKHDE-LTYF- 98
Karlodinium          GGKSESDAFSF--- 115
Nannochloropsis      GGKTLEDAL----- 101
                      .    :
```

Supplementary Figure 4
