# Supplementary material for: Phylogenomic analysis of the cystatin superfamily in eukaryotes and prokaryotes
Source: BMC Evol Biol. 2009 Nov 18;9:266. doi: 10.1186/1471-2148-9-266 (PMC2784779; doi:10.1186/1471-2148-9-266)
Supplement: Additional file 10 — Supplementary Table 6. Ancestral states for the cystatin superfamily in eukaryotic supergroups. [file 1471-2148-9-266-S10.PDF]

Supplementary Table 6. Ancestral states for the cystatin superfamily in eukaryotic supergroups.

Eukaryotic supergroup	stefins	cystatins
Excavata	■	■
SAR supergroup (Stramenopiles + Alveolates + Rhizaria)	■	■
Haptophyta + Cryptophyta	■	■
Archaeplastida	■	■
Amoebozoa	■	■
Opisthokonta	■	■
Unikonta	■	■
Bikonta	■	■
Eukaryota	■	■

Presence is marked with the black square.
