# Supplementary material for: Phylogenomic analysis of the cystatin superfamily in eukaryotes and prokaryotes
Source: BMC Evol Biol. 2009 Nov 18;9:266. doi: 10.1186/1471-2148-9-266 (PMC2784779; doi:10.1186/1471-2148-9-266)
Supplement: Additional file 11 — Supplementary Figure 5. Functionally important structural motif of eukaryotic cystatins is conserved in the bacterial stefins and cystatins. Highly conserved QXVXG region is in bold. Eukaryotic cystatin (Giardia) and stefin (Euglena) have been included. [file 1471-2148-9-266-S11.PDF]

1Vibrio	KAEDKEVFAKGIEGFVGVKYSPLVVAT**QVVAG**QNYAFFCNAEVVYPGAQPYPAMVHMFSD	75
15Vibrio	TAENKEVFAKGIEGFVGVKYSPLVVAT**QVVAG**QNYAFFCNAEVVYPGAQPYPAMVHMFSD	75
13Vibrio	SNEDKAAFASGIEGFVGVSYKPVAVAT**QVVAG**CNYAFFCNAEMVYPGSQPYPAMVHMFKD	103
2Shewanella	DTQAKEAFSEAMEHFVGVDYSPVAVAT**QVVAG**LNYAYFCNAQIPGLGSSVYPAMVNIYKP	76
3Shewanella	NPEAKAAFADAMEHFVGVKYSPVAVAS**QVVSG**TNYSFFCNAEVVVPGSTVYPTMVDVYKP	76
Marinomonas	DERAMMCFKEATAHFDDFIYTPVAVAT**QVVSG**TNYAFFCDVNSKE-SSQVYSAMVTIFKP	72
1Aeromonas	TAEDQAVFDQALKGFVGVQYVPFEVCT**QVVAG**TNYRFKCKSTVPLAKPIHGEAVVQIFQS	77
2Aeromonas	SAKDQAVFNQALEGFVGVQYTPFEVST**QVVAG**TNYRFKCKTTVPLPNPIHGEAVVQIFQS	127
1Shewanella	TSAEREIFNKAMEGFVGVSYMPETVST**QVVAG**MNYRFKCEASMP-PSEVLWEAIVEIYQP	76
3Clostridium	TTYDSFIFYNAIGEHPDYFYRPIAVAK**QVVNG**TNYRFMTIAEPEQSDLTPHFAIVEIYQP	142
4Clostridium	TTYDSFIFYNAIGEHPDYFYRPIAVAK**QVVNG**TNYRFMTIAEPEQSDLTPHFAIVEIYQP	92
Bacteroides	TDQEKEIFRTCMT-LKGVDYTPLLVAK**QLVSG**YNYRFICMTESLIREPKYGFAKVTIYAP	167
2Clostridium	TDDDINVLKTALSELIGVEYEPKILAT**QSVNG**TNYCFICKSKVAAHNGQ-GISKVMIYKP	72
Erwinia	NDTDKALFKSTVH-LLGVTYSPLFVAT**QVVSG**TNYSFLTKGTVTSPEAPLRIVKIHVYKP	74
Burkholderia	TSAEKAVFNAALEGMVGVGYKPLAFAT**QVVAG**TNYCFLCKGQAAFPVAPEFAALIYVNQP	76
Microcystis	TESAKNVFDAALKGFVGVGYTPLAFAT**QVVAG**MNYCFLCKGKVVYPNAPDLVVLVYIYKP	85
2Geobacter	SAQASEVFATATKGLLGVTYTPLAVAT**QVVAG**TNYMFFCNAQVVYPNAPNEAVLMTIFSP	92
Chlorobium	SFTDRVVFRDTMAGLTGVGYEPLVVRK**QIVEG**VNYEFFCNARAVYPGTDWHPAMVLIYKP	165
1Clostridium	EEEDLKVFNEAVGMLKGVDYKPLIVST**QVVAG**TNFKFICNATSVTNPPHNYLAEIIVFKP	74
2Vibrio	QAKQALNAVLGQMNTSAELKQILSVRT**QVVAG**LNYAIEFEMDNGEVWNTVVYRSLQGDIE	118
3Vibrio	QAKQALDVVLGQMNTSAKLKQILSVRT**QVVAG**LNYAIEFEMDNGEVWNTVVYRSLQGDIE	118
Vibrionales	QAEQALDAVLSQMNTSAKLQQILSVRT**QVVAG**LNYAIEFEMDNGEVWNTIVYRSLQGDIE	118
4Vibrio	EAQQALDFVLGQMNTAAKLKEILSVRT**QVVNG**LNYAIEFEMDNGEVWNTIVYRSLKGDME	90
5Vibrio	EAQPLLDVVLAQMNTSAKLKQILSVRT**QVVSG**MNYAIEFEMDNGEIWNTIVYRSLDDEIK	114
1Photobacterium	EATAAVDSVLAMMNTSAQLSKILDVKT**QVVNG**INYAIDFELDNGEVWNTRVYRSLKGKYT	115
2Photobacterium	EATAAVDSVLAMMNTSAQLSKILDVKT**QVVNG**INYAIDFELDNGEVWNTRVYQNLEGKYT	126
6Vibrio	EAKEAVEHVLSMMNTSAKLKQILDVKT**QVVNG**INYAIDFELDDGQVWNTRVYRSLKGQYQ	114
7Vibrio	DAQKAMAFVLKKMETLASFKQILSVHA**QVVSG**VNYAIEFELNDGSVWNTVVYRNLDGEYA	113
10Vibrio	DAQKAMGFVLKKMDTIASFKQILNVHA**QIVSG**VNYAIEFELDDGSVWNTIVYRNLDGEYA	113
14Vibrio	QVMQALDMVLEQMNTNSPLKEVLSAHT**QVVSG**VNYAIEFQLENGSSWNTIVYRNLKGEYQ	113
8Vibrio	EAQRSLSMVLYQMNAEDKLKNINEVRT**QVVAG**THYAFEFELQDGEVWNAMVLRSARGDYM	118
9Vibrio	DAERSLSMILYQMNATDKLKQINDVRT**QVVGG**IHYAMEFELKDGQVWNAIVLHSARGDYM	118
11Vibrio	EVEQAAKDAVKAIPGEHQLGKIYHVTQ**QVVAG**MNYSITFSIENGDYYNATVFRSLQNTFD	112
3Photobacterium	EVEQAAKDAVKAIPGDHQLGKIYHVTQ**QVVAG**MNYSITFSIENGDYYNATVFRSLQNTYD	112
12Vibrio	DVLQAAKYAVKAIPGDHHLGKIYNVKQ**QVVAG**VNYSITFSIENGDYYSAIIFRSLQNTYH	112
4Photobacterium	DVLQAAKYAVKAIPGDHHLGKIYNVKQ**QVVAG**VNYSITFSIENGDYYSAIIFRSLQNTYH	112
Giardia	KVREAAAAKIAESVSGATIAEVIKASS**QVVRG**VNTMLLTRLSTGAHYIVVVWFDLKNYIV	76
1Geobacter	AEQAKVIEKELKTATKLSLVTIISAEE**QVVSG**MNYHLALKVKLNGKRKKAEATVWWQAWN	112
Euglena	KDGVHAAARNTGFAGDFTKYEPVSYKT**QVVAG**TNFFIKLAVAEDQFLHARIFKPLPCNGA	83

Supplementary Figure 5
